# Increased Vulnerability of Human Ventricle to Re-entrant Excitation in hERG-linked Variant 1 Short QT Syndrome

**DOI:** 10.1371/journal.pcbi.1002313

**Published:** 2011-12-15

**Authors:** Ismail Adeniran, Mark J. McPate, Harry J. Witchel, Jules C. Hancox, Henggui Zhang

**Affiliations:** 1 Biological Physics Group, School of Physics & Astronomy, The University of Manchester, Manchester, United Kingdom; 2 Department of Physiology and Cardiovascular Research Laboratories, School of Medical Sciences, Bristol, United Kingdom; University of California San Diego, United States of America

## Abstract

The short QT syndrome (SQTS) is a genetically heterogeneous condition characterized by abbreviated QT intervals and an increased susceptibility to arrhythmia and sudden death. This simulation study identifies arrhythmogenic mechanisms in the rapid-delayed rectifier K^+^ current (I_Kr_)-linked SQT1 variant of the SQTS. Markov chain (MC) models were found to be superior to Hodgkin-Huxley (HH) models in reproducing experimental data regarding effects of the N588K mutation on *KCNH2*-encoded hERG. These ionic channel models were then incorporated into human ventricular action potential (AP) models and into 1D and 2D idealised and realistic transmural ventricular tissue simulations and into a 3D anatomical model. In single cell models, the N588K mutation abbreviated ventricular cell AP duration at 90% repolarization (APD_90_) and decreased the maximal transmural voltage heterogeneity (δV) during APs. This resulted in decreased transmural heterogeneity of APD_90_ and of the effective refractory period (ERP): effects that are anticipated to be anti-arrhythmic rather than pro-arrhythmic. However, with consideration of transmural heterogeneity of I_Kr_ density in the *intact* tissue model based on the ten Tusscher-Noble-Noble-Panfilov ventricular model, not only did the N588K mutation lead to QT-shortening and increases in T-wave amplitude, but δV was found to be augmented in some local regions of ventricle tissue, resulting in increased tissue vulnerability for uni-directional conduction block and predisposing to formation of re-entrant excitation waves. In 2D and 3D tissue models, the N588K mutation facilitated and maintained re-entrant excitation waves due to the reduced substrate size necessary for sustaining re-entry. Thus, in SQT1 the N588K-hERG mutation facilitates initiation and maintenance of ventricular re-entry, increasing the lifespan of re-entrant spiral waves and the stability of scroll waves in 3D tissue.

## Author Summary

Sudden cardiac death may arise in individuals with diseased heart tissue, or in apparently healthy subjects who suffer from genetic defects in ‘ion channel’ proteins, which increase cardiac arrhythmia risk and are associated with significant morbidity and mortality. One rare, though serious, genetic condition is the ‘short QT syndrome’ (SQTS). Although it is now known that the *KCNH2*-encoded N588K-hERG mutation is associated with the main (SQT1) variant of the SQTS, the mechanisms by which ventricular arrhythmia is initiated and sustained are still unclear due to lack of genotypically accurate experimental models. In this study, we used sophisticated multi-scale computer models of human ventricles in order to investigate the pro-arrhythmic effects of the N588K hERG mutation. It was found that the mutation accelerated the ventricular repolarization process, produced augmented electrical heterogeneity in some local regions of the tissue, leading to increased risk of arrhythmia genesis. It was also found that accelerated ventricular repolarization reduced the substrate size of the tissue required to sustain re-entrant circuits in both two and three dimensions. This study provides new mechanistic insight into understanding of how changes to hERG channel function in SQT1 lead to exacerbated ventricular arrhythmia risk in this inherited arrhythmia syndrome.

## Introduction

Impaired cardiac ion channel function can lead to arrhythmias and, thereby, to significant morbidity and mortality. Genetic repolarization disorders include the ‘long’ and ‘short’ QT syndromes in which ventricular repolarization is, respectively, prolonged or accelerated, thereby increasing susceptibility to cardiac arrhythmia [1], [2]. The short QT syndrome (SQTS) was first recognized as a distinct clinical syndrome in 2000 [3]. It is characterised by markedly shortened QT intervals, poor rate adaptation of the QT interval, shortened ventricular and atrial refractory periods, atrial and ventricular arrhythmias and by an increased incidence of sudden death in affected patients [1], [4], [5]. Candidate gene screening has revealed three forms of potassium channel linked SQTS associated with distinct mutations to *KCNH2 (hERG), KCNQ1(KvLQT1)* and *KCNJ2* encoded-potassium channel subunits [6]–[10]. The first identified SQT1 variant is caused by base changes that lead to a common amino-acid substitution (asparagine588lysine; N588K) in the external S5-Pore linker of the hERG potassium channel [7], [8]. This mutation leads to a marked right-ward voltage shift in hERG channel inactivation [11], [12], reflected in action potential (AP) voltage-clamp experiments by a markedly increased hERG current (I_hERG_) during both ventricular and atrial AP repolarization [7], [11]–[13].

As hERG underpins the rapid delayed rectifier channel current (I_Kr_) [14], the N588K ‘gain-of-function’ hERG mutation is anticipated to increase greatly the contribution of I_Kr_ to cardiac repolarization in SQT1 patients, thereby accounting for QT interval abbreviation [7], [11]–[13]. Although this effect would be anticipated to shorten ventricular effective refractory period (ERP) and thereby to increase susceptibility to re-entrant arrhythmia, there is at present no genetically accurate animal model in which the basis of arrhythmogenesis in SQT can be explored. Data from experiments using the perfused canine left-ventricular wedge preparation treated with the K_ATP_ channel opener pinacidil or the I_Kr_ activator PD-118057 suggest roles in the substrate for ventricular tachycardia with QT interval shortening for ERP abbreviation and heterogeneous abbreviation of APs across the ventricular wall (and thereby amplified dispersion of repolarization) [15], [16]. However, neither of these pharmacological interventions reproduces precisely the changes to I_Kr_ caused by the N588K hERG SQT1 mutation. An alternative approach to determine the arrhythmogenic substrates in the SQTS is *in silico* reconstruction of activity in the absence and presence of SQT mutations [10], [17]–[20]. In the case of SQT1, the available simulation data regarding functional consequences of the SQT1 mutation are either incomplete or based on data obtained at ambient rather than physiological temperature [10], [17], [18], [20]. Moreover, no simulation study has yet demonstrated a viable *tissue* substrate for ventricular arrhythmia in SQT1. Accordingly, the present study was conducted in order: (i) to recapitulate the kinetic changes to I_Kr_ in SQT1 based on available experimental data at physiological temperature by utilising and comparing Hodgkin-Huxley and Markov chain formulations; (ii) using human ventricular cell-based models to determine the functional consequences of incorporating the SQT1 mutation on AP repolarization and the QT interval; (iii) to explore the arrhythmogenic substrate in SQT1 by using “idealized” and “realistic” 2D tissue and 3D organ simulations. The results of our study provide a clear link between the kinetic changes to I_hERG_/I_Kr_ in SQT1 and altered ventricular tissue electrophysiology that favours re-entrant arrhythmia in this form of the SQTS.

## Results

### Simulation of single cell I_Kr_ under control and SQT1 conditions

**Figure 1:**
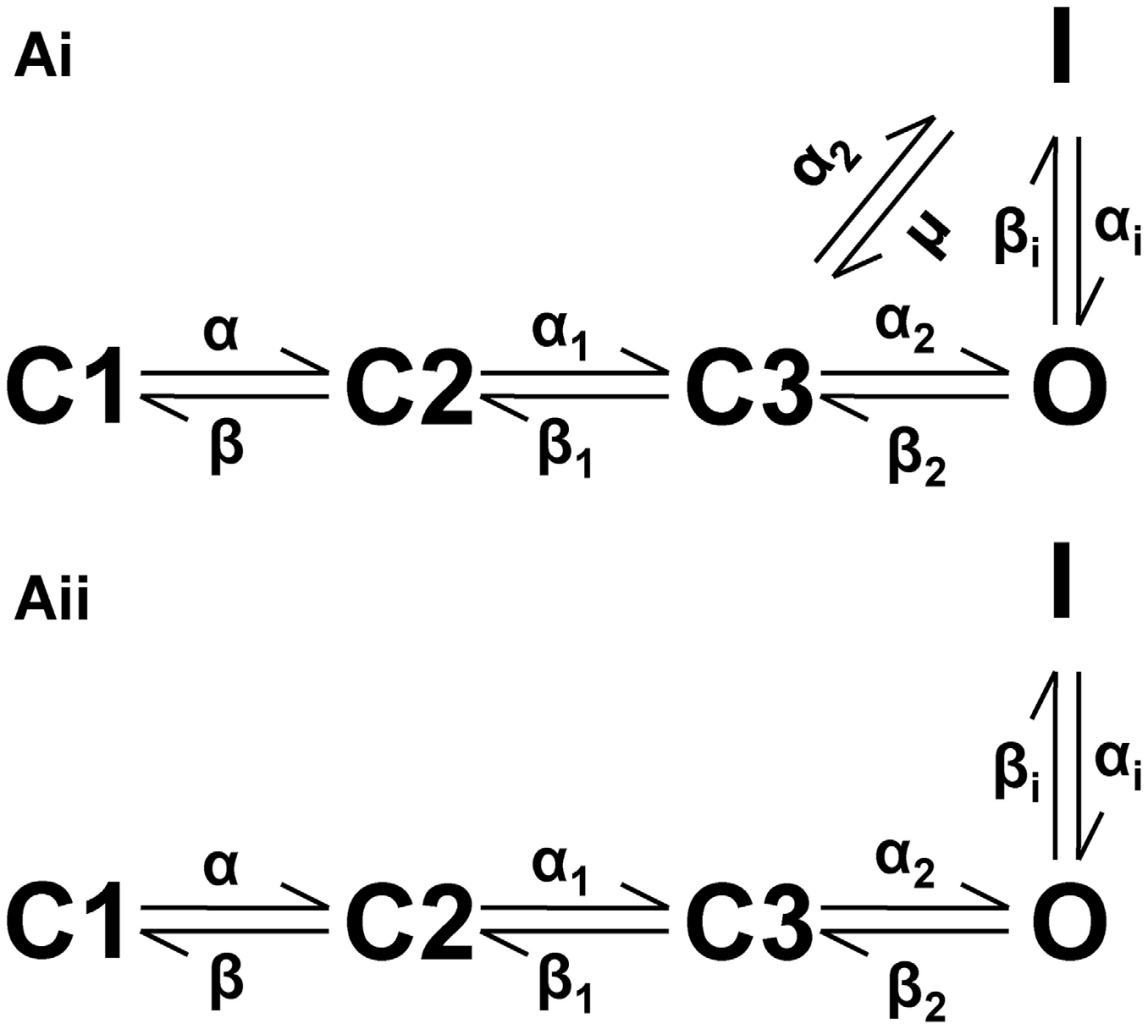
State transition diagrams of the Markov models. (Ai) Full Markov Chain (f-MC) state transition diagram. (Aii) Reduced Markov Chain (r-MC) state transition diagram.

**Figure 2:**
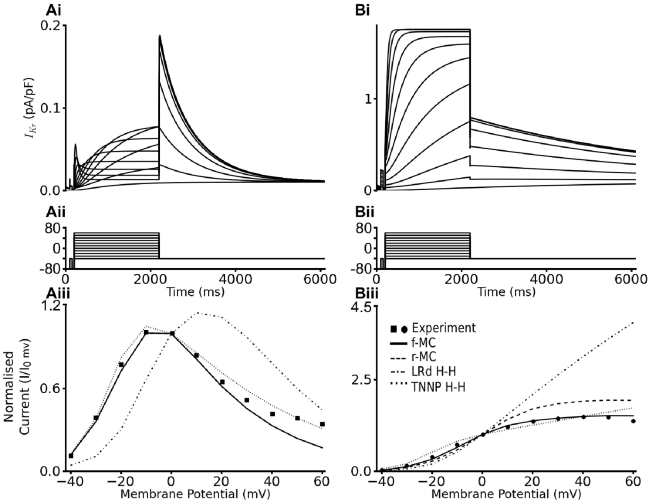
Simulated Current-Voltage Relationships for I_hERG_. (**Ai, Bi**) Current traces for WT (A) and N588K I_hERG_/I_Kr_ (B) elicited by the voltage protocol shown in (**Aii, Bii**). (**Aiii, Biii**) I-V relation for end pulse currents for WT (A) and N588K I_hERG_/I_Kr_ (B). End pulse currents were normalised to the current observed at 0 mV and then plotted against membrane potential.

We first tested the ability of the developed full Markov chain (f-MC) I_Kr_ model (see Figure 1A for the gating scheme) to reproduce previously published experimental data [12], [13] on the voltage dependence of WT and N588K I_hERG_ at physiological temperature. The voltage-clamp protocol used (Figure 2Aii and 2Bii) was the same as that used experimentally [12]. Figure 2 illustrates the profile of outward I_Kr_/I_hERG_ under the WT (Figure 2Ai) and SQT1 mutation (Figure 2Bi) conditions, from which the corresponding I-V relationships were reconstructed (see Methods) and shown in Figure 2Aiii & Biii respectively. For both WT and mutation conditions, the I-V relationships reconstructed from I_Kr_ during simulated voltage-clamp matched those seen experimentally. Most significantly, the f-MC model recaptured the features of the mutation-induced defect of I_Kr_ inactivation, which led to augmented I_Kr_ current as has been observed experimentally for recombinant hERG channels at 37°C [12], [13]. With the same voltage-clamp protocol, the I-V relationship simulated from the reduced Markov chain (r-MC) model (see Figure 1B) also matched prior experimental data. Both the f-MC and the r-MC models were able to recapture the voltage-dependence of I_Kr_ better than the Hodgkin- Huxley (H-H) formulations with either the Luo-Rudy or the TNNP equations as shown in Figure 2 for either the WT (Figure 2Aiii) or the mutation (Figure 2Biii) condition.

**Figure 3:**
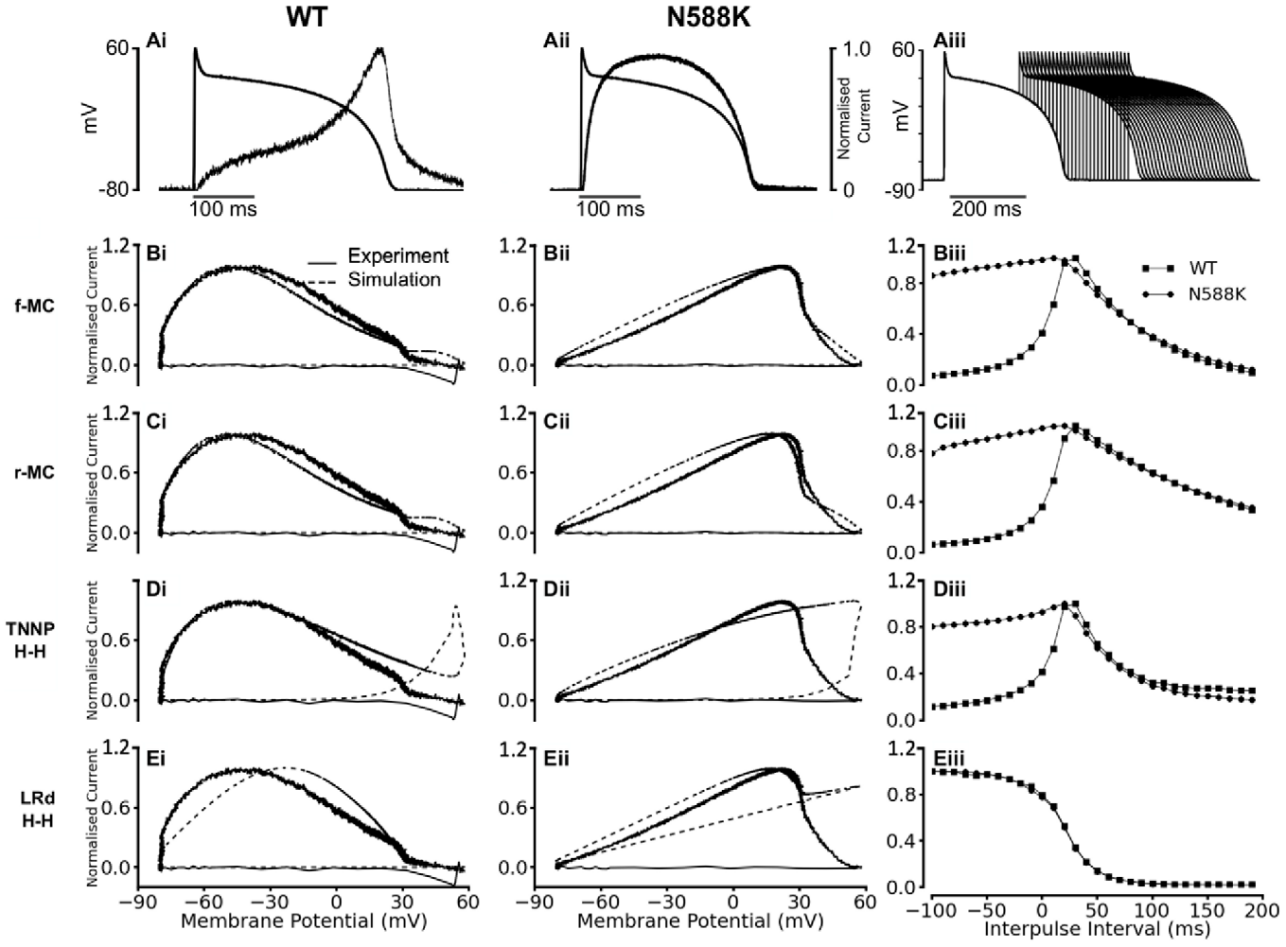
I_hERG_/I_Kr_ Current-Voltage (I-V) relations during action potential clamp and effect of premature stimuli. (**A**) Current profiles of WT (Ai) and N588K I_hERG_ (Aii) elicited by the ventricular AP command wave form overlaid. In each case, instantaneous current during AP repolarisation was normalised to maximal current elicited by the waveform. (Aiii) Paired ventricular AP command waveform protocol used to elicit the I_hERG_/I_Kr_ currents from which the normalized data in Biii, Ciii, Diii and Eiii were derived. (**B**) Full Markov chain model: Instantaneous I-V relationships for WT (Bi) and N588K I_hERG_/I_Kr_ (Bii). Thick lines show experimental recordings while the dashed lines show simulation results. (Biii) Plots of theI_hERG_/I_Kr_current during paired AP command waveforms (Aiii) for WT (squares) and N588K condition (circles) respectively. (**C**) Reduced Markov chain model: Instantaneous I-V relationships for WT (Ci) and N588K I_hERG_/I_Kr_ (Cii). Thick lines show experimental recordings while the dashed lines show simulation results. (Ciii) Plots of I_hERG_/I_Kr_ current during paired ventricular AP command waveforms (Aiii) for WT (squares) and N588K condition (circles) respectively. (**D**) ten Tusscher *et al.* model: Instantaneous I-V relationships for WT (Di) and N588K I_hERG_/I_Kr_ (Dii). Thick lines show experimental recordings while the dashed lines show simulation results. (Diii) Plots of I_hERG_/I_Kr_ current during paired ventricular AP command waveforms (Aiii) for WT (squares) and N588K condition (circles) respectively. (**E**) Luo-Rudy model: Instantaneous I-V relationships for WT (Ei) and N588K I_hERG_/I_Kr_ (Eii). Thick lines show experimental recordings while the dashed lines show simulation results. (Eiii) Plots of I_hERG_/I_Kr_ current during paired ventricular AP command waveforms (insert in Eiii) for WT (squares) and N588K condition (circles) respectively.

We then examined the ability of the developed I_Kr_ models to reproduce the dynamic properties of WT and N588K mutation I_hERG_/I_Kr_ channels, by using simulated action potential (AP) voltage clamp, incorporating paired AP commands [13]. Figure 3 shows the simulated normalised ‘instantaneous’ I-V relationship for current during the time course of AP clamp under WT (Figure 3Ai–Di) and the mutation (Figure 3Aii–Dii) conditions. It also shows the time-course of outward current “transients” elicited by a protocol comprised of paired AP commands (Figure 3Aiii–Diii). Simulation data from the f-MC, r-MC and the H-H models were compared with published experimental ventricular AP clamp data obtained from CHO cells with expression of WT and N588K hERG channels [13]. During the time course of the AP, both the f-MC and r-MC I_Kr_ models reproduced experimental instantaneous I-V data for the WT (Figure 3Ai & Bi) and mutation (Figure 3Aii & Bii) conditions, as well as the positive shift in the peak repolarizing current caused by the N588K mutation [13] (Figure S1 in Text S1). However, the simulated I-V relation from the H-H I_Kr_ formulations of the TNNP model (Figure 3Ci–Cii) or the Luo-Rudy model (Figure 3Di–Dii) did not closely match experimental data, for either the WT or mutation conditions (Figure S2 in Text S1). Responses of hERG/I_Kr_ to a protocol comprised of paired-AP stimuli [13] are shown in the right panels. Each of the f-MC, r-MC and TNNP H-H models reproduced the response of hERG/I_Kr_ channel to a premature stimulus as seen experimentally for both the WT and the mutation condition [13], which reflects the interaction between recovery from inactivation and deactivation of I_hERG_/I_Kr_ channels [13], [21] (Figure 3Aiii–Ciii) under both conditions. For the WT condition, the amplitude of I_Kr_ increased with the increase of the inter-pulse interval, and reached a maximal amplitude at 30 ms before decreasing at greater time intervals. For the N588K condition, I_Kr_ amplitude peaked 10 ms earlier, and was significantly greater at small intervals but decreased more extensively than WT between 30–70 ms of the inter-pulse interval. However, the response of the Luo-Rudy H-H model to paired-AP stimuli did not reproduce experimental data (Figure 3Diii). Considered collectively, the AP clamp simulation data suggested that the f-MC and the r-MC models recapitulated better the dynamic properties of WT and N588K I_hERG_/I_Kr_ at 37°C than did the TNNP and Luo-Rudy H-H formulations tested in this study.

**Figure 4:**
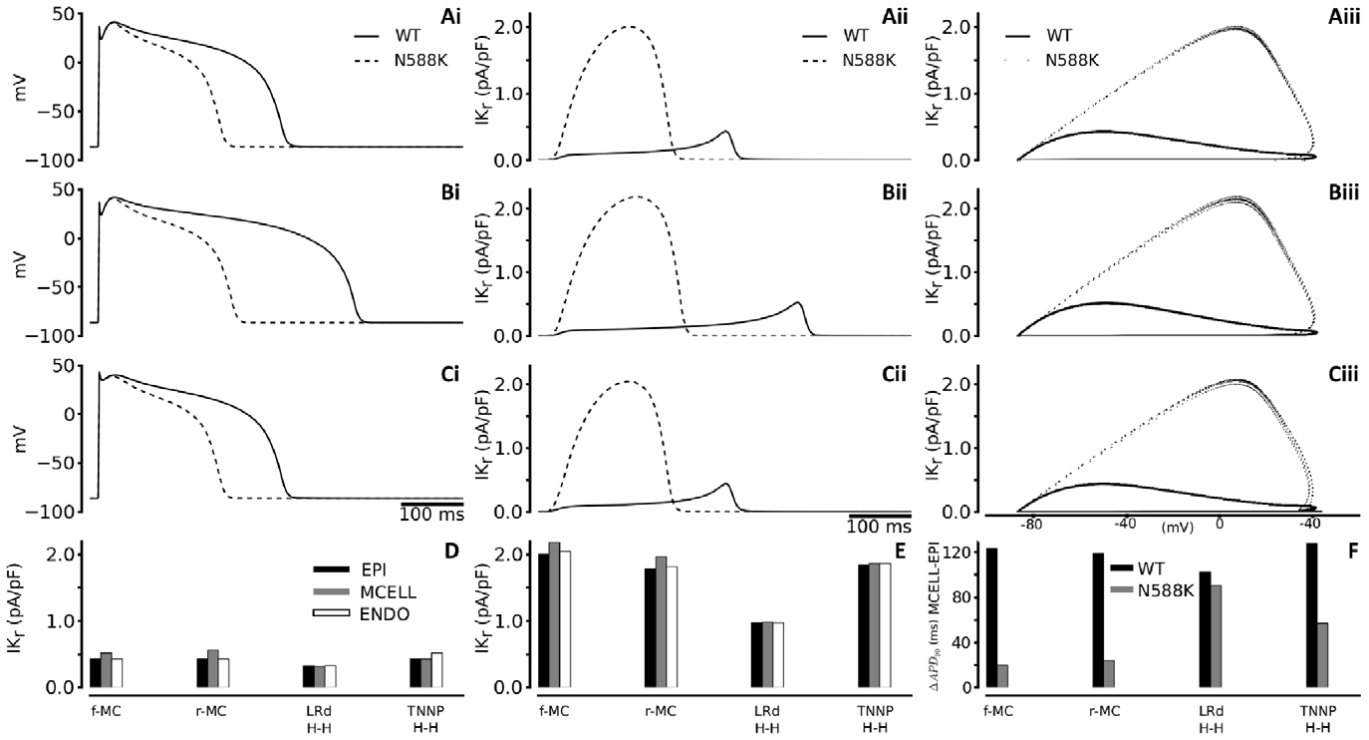
Simulation of action potential and I_Kr_ time courses. (**i**) Steady state (1 Hz) action potentials for EPI (Ai), MIDDLE (Bi) and ENDO (Ci) cells using the full Markov chain I_hERG_/I_Kr_ model. Thick lines represent WT and dashed lines represent N588K condition. (**ii**) Corresponding I_Kr_ current profiles for EPI (Aii), MIDDLE (Bii) and ENDO (Cii) cells. Thick lines represent WT and dashed lines represent N588K condition. (**iii**) Corresponding I-V relationships for EPI (Aiii), MIDDLE (Biii) and ENDO (Ciii) cells. Thick lines represent WT and dotted lines represent N588K condition. (**D,E**) I_Kr_ current amplitude for EPI (black bars), MIDDLE (grey bars) and ENDO (white bars) cells for all four I_hERG_/I_Kr_ formulations for WT (D) and N588K (E) conditions (**F**) Computed APD difference between EPI and MIDDLE cells.

The functional effects of the N588K mutation on ventricular APs were characterised by using modified TNNP models that incorporated the f-MC, r-MC and both H-H I_Kr_ formulations (the original TNNP I_Kr_ equations and the Luo-Rudy I_Kr_ equations) for the WT and mutation conditions. Figure 4A shows simulated APs (Ai), I_Kr_ profile (Aii) and I_Kr_ instantaneous I-V relationship (Aiii) of an EPI cell model with the f-MC I_Kr_. WT I_Kr_ increased progressively during the time course of initial AP upstroke and later AP plateau phases, reaching a maximal amplitude prior to a rapid decrease during terminal AP repolarization. With the N588K mutation, I_Kr_ increased more rapidly following the AP upstroke and reached a significantly higher peak amplitude, leading to marked APD abbreviation (APD_90_ changes are summarised in Table 1).

**Table 1:** Action potential duration and differences for the WT and N588K conditions for all four I_hERG_/I_Kr_ model formulations.

Model	Cell Type	WT	N588K	ΔAPD_90_ (ms)
		APD_90_ (ms)	APD_90_ (ms)	
**f-MC**	**EPI**	317	212	105
	**MCELL**	441	232	209
	**ENDO**	317	211	106
**r-MC**	**EPI**	317	225	92
	**MCELL**	436	249	187
	**ENDO**	317	224	93
**TNNP**	**EPI**	315	245	70
	**MCELL**	443	302	141
	**ENDO**	315	246	69
**Luo-Rudy**	**EPI**	316	295	21
	**MCELL**	419	386	33
	**ENDO**	316	296	20

Computed APD_90_ (ms) and ΔAPD_90_ (ms) under WT and N588K condition for the ENDO, MIDDLE and EPI cell models. ΔAPD_90_ was computed as the difference of APD_90_ between that of the control condition and that of the N588K condition.

Such APD abbreviation was attributable to both the I_Kr_ amplitude increase and altered timing of the current, with peak I_Kr_ occurring earlier during the AP plateau (Figure 4Aii). Simulation results with the MIDDLE and ENDO cell models were similar in showing a marked APD reduction as illustrated in Figure 4B & 4C. Results for each of the f-MC, r-MC and H-H I_Kr_ formulations in the EPI, MIDDLE and ENDO cell models are summarised in Figure 4D–4F. This set of simulations with variant I_Kr_ formulations in all cell models showed marked APD reduction associated with increased I_Kr_ by the N588K mutation, which correlates well with previous studies in which a gain in I_Kr_ due to N588K mutation produced AP shortening in the Luo-Rudy and the Priebe-Beuckelmann AP models [17], [18], [20]. The alteration to APD with the N588K mutation was non-uniform, with the largest reduction occurring for the MIDDLE cell [18], [20]. As a consequence, the APD_90_ difference between ENDO-MIDDLE-EPI cells was attenuated by the mutation, leading to a decreased transmural dispersion of APD_90_ when the three cell models were compared (Figure 4F). Simulation results with the uses of f-MC, r-MC and H-H formulations all gave consistent results of attenuated APD_90_ dispersion by the mutation when the three cell models were compared (Figure 4F).

**Figure 5:**
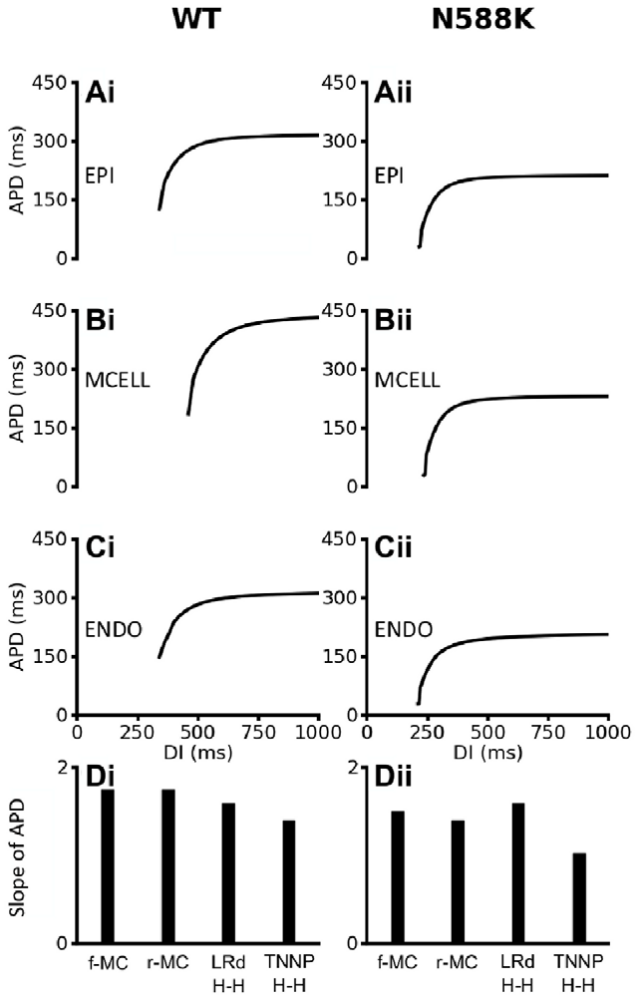
Rate-dependent APD restitution curves of four models of ventricular myocytes. (**Ai, Bi, Ci**): WT APD restitution curves for EPI, MIDDLE and ENDO cells respectively for the full Markov chain I_hERG_/I_Kr_ model formulation. (**Aii, Bii, Cii**) N588K APD restitution curves for EPI, MIDDLE and ENDO cells respectively for the full Markov chain I_hERG_/I_Kr_ model formulation. (**D, Dii**) Slopes of WT and N588K APD restitution curves for full Markov chain, reduced Markov chain, Luo-Rudy and TNNP hERG/I_Kr_ model formulation.

The N588K mutation flattened the APD restitution curves as shown in Figure 5. For each of the three cell models, the four different I_Kr_ formulations were adjusted to produce similar APD_90_ restitution curves under the WT condition for EPI, MIDDLE and ENDO cells (data shown only for the f-MC formulation in Figure 5A–C). Incorporation of the N588K mutation decreased the measured APD_90_, causing a leftward shift to the APD restitution curve. The maximal slopes of the APD restitution curves for the Markov and TNNP HH formulations were also reduced (Figure 5D). These results suggested an attenuation of rate-adaptation of ventricular APD, which is consistent with the poor rate-dependence of QT intervals in SQT patients. This was investigated further by plotting the steady-state APD-rate dependence for WT and N588K conditions (Figure S3 in Text S1 for the TNNP model with the f-MC I_Kr_ formulation); the relationship was flattened and leftward shifted in the setting of SQT1. Among the four different I_Kr_ formulations, the mutant f-MC and r-MC I_Kr_ models produced significantly greater APD abbreviation than the H-H formulations of the Luo-Rudy and the TNNP I_Kr_ models.

**Figure 6:**
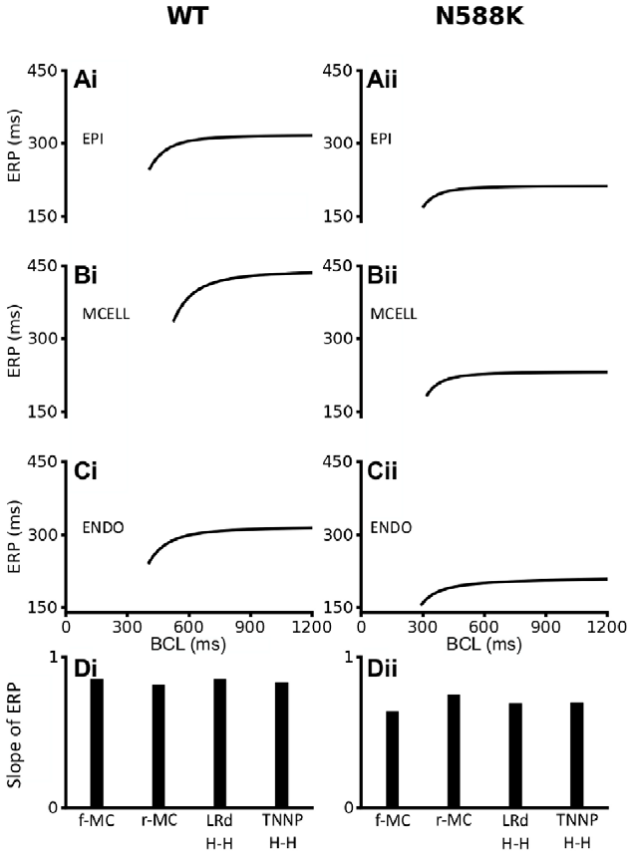
ERP restitution curves of four models of ventricular myocytes. (**Ai, Bi, Ci**) WT ERP restitution curves for EPI, MIDDLE and ENDO cells respectively for the full Markov chain I_hERG_/I_Kr_ model formulation. (**Aii, Bii, Cii**) N588K ERP restitution curves for EPI, MIDDLE and ENDO cells respectively for the full Markov chain I_hERG_/I_Kr_ model formulation. (**Di, Dii**) Slopes of WT and N588K ERP restitution curves respectively for full Markov chain, reduced Markov chain, Luo-Rudy and TNNP I_hERG_/I_Kr_ model formulations.

Simulation of the N588K mutation also abbreviated the effective refractory period (ERP) for all TNNP ventricular cell models, as shown in Figure 6. Results from all of the three cell models with variant I_Kr_ formulations (shown in Figure 6A–C for the f-MC model) consistently showed that the mutation abbreviated ventricular ERP and caused a leftward shift in the ERP restitution curves with decreased maximal slopes (Figure 6Dii). These results also suggested a loss of rate-adaptation of ventricular ERP. For comparative purposes, the f-MC WT and N588K I_Kr_ formulations were inserted into two other human ventricular cell models, the Grandi-Pasqualini-Bers (GPB) [22] and O'Hara-Rudy dynamic (ORd) [23] models. The results are shown in Figure S4 in Text S1 and Figure S5 in Text S1 and indicate that at the single cell level, the functional consequences of the N588K mutation (abbreviated APD, decreased APD heterogeneity, flattened APD restitution and rate dependence) are model-independent.

### Simulation of the ECG with WT and SQT1 mutant I_Kr_

**Figure 7:**
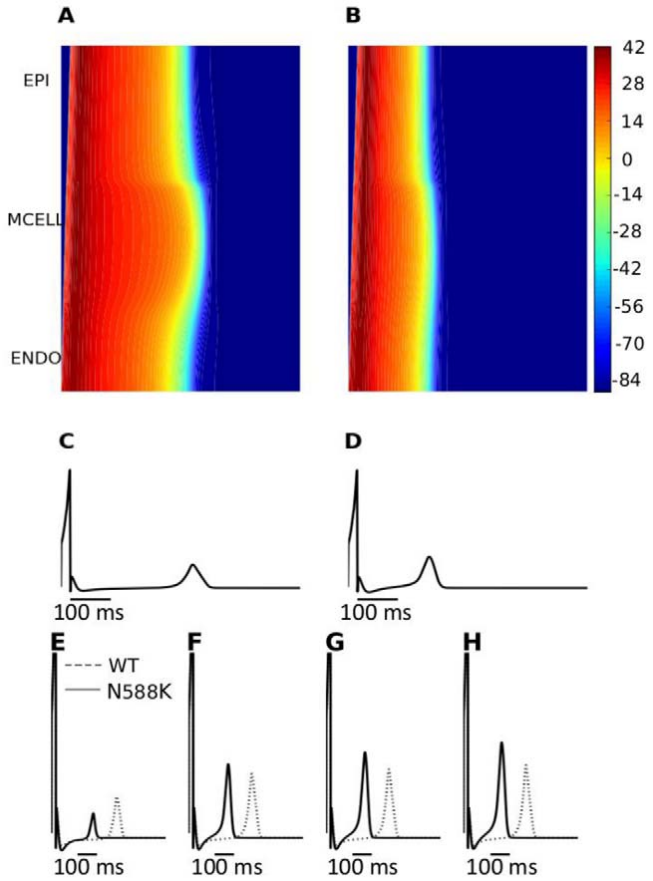
Space-time plot of AP propagation along a 1D transmural ventricular strand and computed pseudo-ECGs. (**A, B**) Colour mapping of membrane potential of cells along the 1D strand from blue (−86 mV) to red (−42 mV) (see colour key). Space runs vertically from the ENDO end to the EPI end at the top. Time runs horizontally. (A) Control (WT) condition. (B) SQT1 (N588K) condition. (**C, D**) Pseudo-ECGs corresponding to the WT and SQT1 (N588K) conditions respectively. (**E, F, G and H**) WT and N588K pseudo-ECGs for the different EPI∶ MIDDLE∶ENDO I_Kr_ density ratios of 1.0∶1∶1, 1.5∶1∶1, 1.6∶1∶1 and 1.7∶1∶1 respectively.

Using a 1D-strand model of tissue across the ventricular wall a pseudo-ECG was computed for the WT and N588K mutation conditions at a stimulation rate of 1 Hz. The f-MC model was used for these and subsequent simulations. The results are shown in Figure 7A–D. In our initial simulations, the computed QT interval was shortened from 378 ms in the control condition to 240 ms as a consequence of the N588K mutation. The abbreviated QT interval in the simulations reproduces a key feature of the ECGs recorded from SQT1 patients – namely QT interval shortening. However, the model failed to reproduce another major feature of SQT ECG: a significant increase in the T-wave amplitude (Figure 7E). To solve this discrepancy, it transpired to be necessary to incorporate a heterogeneous I_Kr_ density in the 1D strand model. This is concordant with some available experimental data, as it has been observed that hERG mRNA expression is about 1.6 times more abundant in the EPI region than in the MIDDLE region from all three cell models [24], consistent with possible transmural heterogeneity of I_Kr_ density. The original TNNP EPI, MIDDLE and ENDO models assume a uniform I_Kr_ density. However, when EPI I_Kr_ was set to be 1.5–1.7 times greater than that in the MIDDLE and EPI cells, the 1D strand model was then able to produce both QT interval abbreviation and an increased T-wave amplitude with the N588K mutation (Figure 7F–7H), matching both major features of SQT ECGs [7], [25]–[27]. Simulation results with the r-MC I_Kr_ model were similar to those with the f-MC formulation under both the WT and the mutation conditions.

For comparative purposes, additional 1D strand simulations were performed using the GPB [22] and ORd [23] ventricle models. The results with these models (Figure S6 in Text S1) indicate that, although all 3 ventricular cell models produced qualitatively similar effects on AP parameters at the single cell level, at the 1D strand level only with the TNNP model did incorporation of SQT1 mutant I_Kr_ lead to increased upright T-wave amplitude, and that this required transmural heterogeneity in I_Kr_ expression (more details are presented in Text S1).

**Figure 8:**
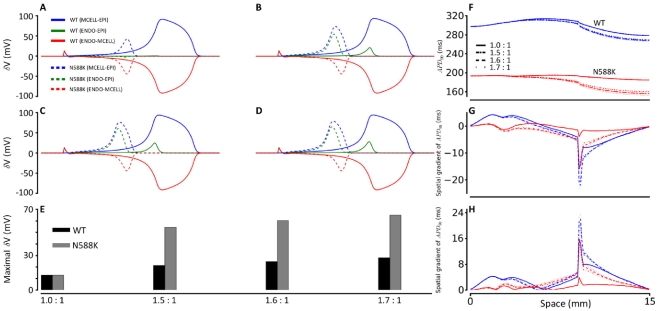
Membrane potential heterogeneity (*δV*), transmural APD_90_ distribution and its spatial gradient along a 1D transmural strand.

In order to examine the factors responsible for an increased T-wave amplitude in the SQT1 setting with the TNNP ventricle model, we examined the effects of the N588K mutation on the membrane potential heterogeneity (δV) between the EPI, MIDDLE and ENDO cells, as well as transmural APD dispersion across the intact 1D strand. Results are shown in Figure 8. In Figure 8A–D, the pairwise differences of membrane potential (δV) during a single AP cycle between EPI, MIDDLE and ENDO cells are shown for differing ratios of EPI I_Kr_ to MIDDLE and ENDO I_Kr_ in the WT (solid lines) and the N588K (dotted lines) mutation conditions. With a 1∶1∶1 ratio (Figure 8A) of EPI I_Kr_ to MIDDLE and ENDO I_Kr_, the N588K mutation decreased the δV in each pairwise comparison. However, with 1.5∶1∶1 (Figure 8B) or a higher (1.6∶1∶1 Figure 8C; 1.7∶1∶1 Figure 8D) ratio of the EPI I_Kr_ to MIDDLE and ENDO I_Kr_, the N588K mutation increased the δV (Figure 8E), which contributed to the increased T-wave amplitude [19], [28]. In the intact 1D strand, the electronic interactions between cells smoothed out the APD distribution as shown in Figure 8F for both the WT and the mutation conditions. With a 1∶1∶1 ratio of EPI I_Kr_ to MIDDLE and ENDO I_Kr_, the mutation attenuated APD dispersion across the strand as shown by the plotted APD spatial gradient (Figure 8G) and its absolute value (Figure 8H). However, with a ratio of 1.5 or above for the EPI I_Kr_ to MIDDLE and ENDO I_Kr_, the N588K mutation augmented APD dispersion at localised regions of the MIDDLE region and at the junction region between the MIDDLE and EPI regions, which also contributed to an increased T-wave amplitude.

### Investigating the arrhythmogenic substrate in SQT1 – 1D simulations

**Figure 9:**
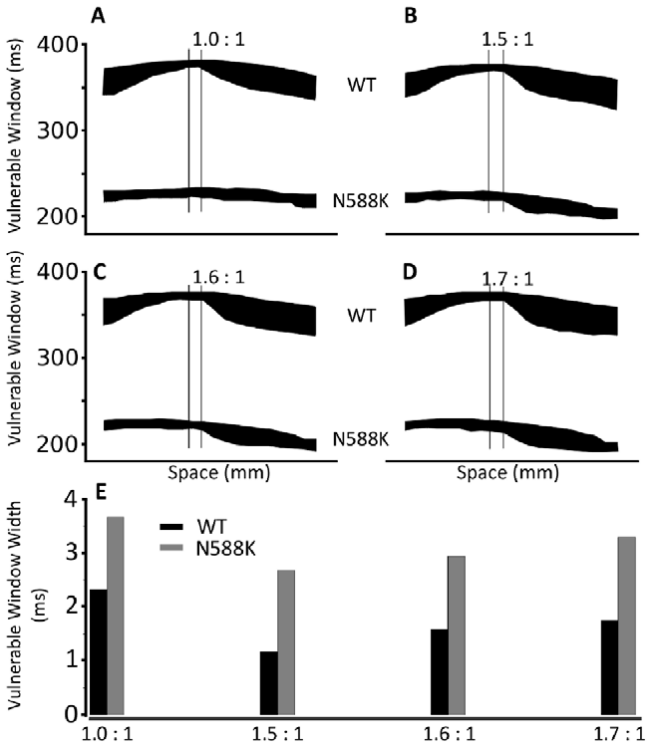
Measured width of vulnerable window along the 1D strand. (**A, B, C, D**) Vulnerable window for WT and N588K along the 1D strand for different EPI∶MIDDLE∶ENDO I_Kr_ density ratios; (A) 1.0∶1∶1 (B) 1.5∶1∶1 (C) 1.6∶1∶1 (D) 1.7∶1∶1. (**E**) Comparison of the width of the vulnerable window between WT and N588K in the MIDDLE region of the 1D strand marked by double lines.

Using 1D-tissue strand simulations with the TNNP model we investigated the vulnerability of tissue to unidirectional conduction block, in response to a premature stimulus applied to the refractory tail of a previous excitation wave. Such vulnerability provides an index of the risk of generating re-entrant excitation in response to a premature stimulus that leads to fibrillation. Results are shown in Figure 9 for the measured width of the vulnerable time window across the transmural strand under the WT and the N588K mutant conditions, during which a premature stimulus provoked a uni-directional conduction block. It was shown that the mutation decreased the measured width of the vulnerable window throughout the majority of the strand. However, at the site in the MIDDLE region marked by the superimposed vertical lines, the measured width of the vulnerable window was increased by the mutation as shown in Figure 9E. These results indicate a localised increase in tissue vulnerability to arrhythmia in the setting of the N588K mutation.

### Investigating the arrhythmogenic substrate in SQT1 – idealised 2D geometry simulations

**Figure 10:**
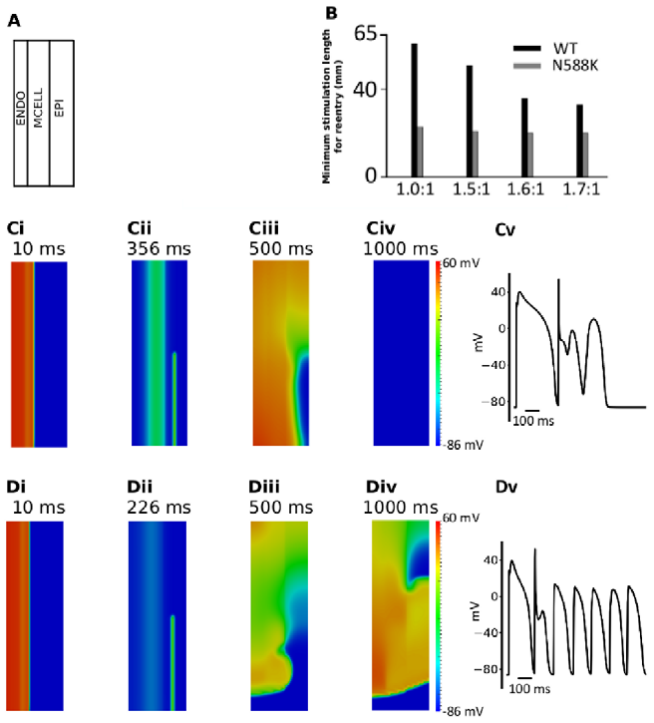
Snapshots of initiation and conduction of re-entry in a 2D idealised model of transmural ventricle. (**A**) Schematic representation of the 2D model. (**B**) Measured minimal spatial length of a premature S2 stimulus that provides a sufficient substrate for the formation of a re-entrant circuit in WT and N588K for different EPI∶ MIDDLE∶ENDO I_Kr_ density ratios; 1.0∶1∶1, 1.5∶1∶1, 1.6∶1∶1 and 1.7∶1∶1. (**C, D**) Ci and Di: A planar conditioning wave generated by S1 stimulus at the ENDO end, which propagates towards the EPI end. Snapshots at time = 10 ms. Cii and Dii: S2 stimulus applied to the EPI part during the vulnerable window of the local tissue Ciii and Diii: Developed spiral wave from the S2 stimulus. Snapshots at time = 500 ms. Civ and Div: Snapshot of spiral wave at time = 1000 ms. Spiral wave self-terminated under the control condition before this recording point, but persisted under the SQT1 condition. Cv and Dv: Evolution of the action potential of a cell in the epicardial region for WT and N588K conditions.

Using a 2D idealised tissue model with contiguous regions of distinctive cell types shown in Figure 10A, we proceeded to measure the minimal spatial size of a premature test stimulus during the vulnerable window that produced uni-directional conduction, leading to genesis of re-entry (spiral wave) for WT and N588K mutation conditions (Figure 10 and Videos S1, S2, S3, S4, S5, S6, S7, S8) with 1.6∶1∶1 of the EPI I_Kr_ to MIDDLE and ENDO I_Kr_. During the simulation, a conditional stimulus (with varying lengths) was applied to the ENDO side of the 2D model to evoke a planar wave that propagated towards the MIDDLE and EPI regions for the WT (Figure 10Ci) and N588K mutant (Figure 10Di) conditions. After a time delay, a premature stimulus was applied to a localised EPI region (Figure 10Cii for WT and Figure 10Dii for N588K), that produced an unidirectional conduction towards the EPI side due to a longer refractory period of the MIDDLE, forming a re-entrant excitation wave (Figure 10Ciii for WT; Figure 10Diii for N588K). The induced re-entrant excitation wave self-terminated for the WT condition (Figure 10Ciiii), but was sustained for the N588K mutation condition (Figure 10Diiii). In both the WT and mutant conditions, the formation of the induced re-entrant excitation wave was dependent on the size of the premature stimulus. We therefore measured the minimal size of the premature stimulus that enabled the formation of re-entry, as this is correlated with the wavelength of excitation (the product of APD and conduction velocity) and measures the minimal size of ventricular substrate necessary to sustain re-entry. For the WT condition, the measured size was large (51 mm) (Figure 10B) due to a comparatively long effective refractory period. Once initiated, the re-entry self-terminated within 284 ms. However, as the N588K mutation shortened the ERP and therefore the wavelength of the ventricular excitation wave, the size of the substrate was reduced to 23 mm (Figure 10B). Once initiated, the spiral wave was found to be sustained. As the minimal substrate size quantifies (in a reciprocal fashion) the tissue's spatial vulnerability to sustain re-entry, this reduction demonstrated an increased tissue susceptibility to arrhythmia in the setting of the N588K mutation.

### Investigating the arrhythmogenic substrate in SQT1 – 2D and 3D simulations with more realistic geometry

Due to the complex geometry and anisotropic properties of ventricular tissue, it cannot be assumed that sustained re-entry in a 2D idealised tissue model under SQT1 conditions necessarily translates into similar activity in a more realistic tissue model. Therefore, further simulations were performed in a 2D human ventricle tissue slice and with human 3D anatomical ventricle geometry. Both the 2D and 3D anatomical models represent not only realistic geometries of left and right human ventricles, but also anisotropic conduction due to fibre orientations. The heterogeneous cellular electrical properties of EPI, MIDDLE and EPI regions were also considered.

**Figure 11:**
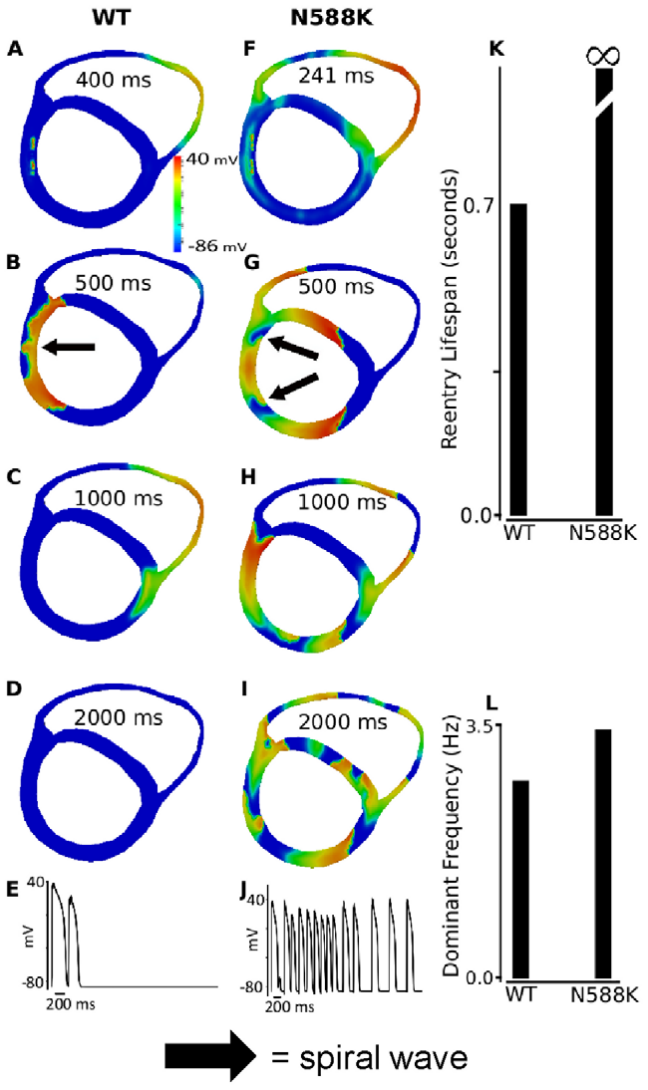
Snapshots of initiation and conduction of re-entry in realistic 2D model cross-section of ventricles. (**A, F**) Application of a premature S2 stimulus into the refractory and partially recovered region of an excitation wave after a delay of 400 ms for WT and 241 ms for N588K condition from the initial wave stimulus. (**B, G**) Developed spiral wave from the S2 stimulus. Snapshot at time = 500 ms. (**C, H**) Snapshot of spiral wave at time = 1000 ms. The induced spiral wave transited from transmural re-entry with tip rotating within the ventricle wall to anatomical re-entry with tip rotating around the ventricle boundary in WT. However, transmural re-entry persisted in N588K condition and broke-up forming regenerative multiple re-entrant wavelets. (**D and I**) Snapshot of spiral wave at time = 2000 ms. Spiral wave self-terminated in WT before this recording point, but persisted in N588K condition. (**E and J**) Evolution of the action potential of a cell in the left ventricle for WT and N588K conditions. (**K**) Measured lifespan of the re-entry circuits in WT and N588K condition. (**L**) Computed dominant frequency of electrical activity recorded from the tissue in WT and N588K condition (about 2.7 Hz for WT and 3.4 Hz for N588K condition).

Results of 2D simulations with realistic human ventricle geometry are shown in Figure 11 (and Videos S1, S2, S3, S4, S5, S6, S7, S8) with 1.6∶1∶1 of the EPI I_Kr_ to MIDDLE and ENDO I_Kr_. In responding to a localised premature stimulus applied in the endocardium, (marked by arrow) within the vulnerable window (WT: 400 ms after the arrival of conditional wavefront; N588K: 241 ms after the arrival of conditional wavefront), a re-entrant excitation wave was initiated within the left ventricular wall as shown in Figure 11A for the WT and Figure 11F for the mutation conditions. Subsequent conduction of the induced re-entrant excitation wave is shown by snapshots included in Figure 11B–D for the WT and Figure 11G–I for the N588K mutation conditions. For the WT condition, the initiated re-entry self-terminated after 755 ms (Figure 11D). However, with the N588K mutation, re-entry was sustained throughout the whole simulation period (5 s) (Figure 11K). Figures 11E and 11J show a recording of the evolution of the AP of a cell in the left ventricle for the WT and N588K conditions. Power spectrum analysis on the recorded action potentials from a localised site (dashed arrow) of the tissue illustrated a higher dominant frequency in the mutation condition, as compared to the WT condition (Figure 11L). An S2 stimulus was also applied in the epicardium to initiate re-entry. The results (not shown) were similar to those obtained when re-entry was initiated by an endocardial stimulus.

The results from this set of simulations are consistent with the simulation results shown in Figure 10 with an idealised 2D ventricular tissue model, thereby further supporting the notion that the N588K mutation increased tissue susceptibility to the genesis and maintenance of re-entrant excitation waves.

**Figure 12:**
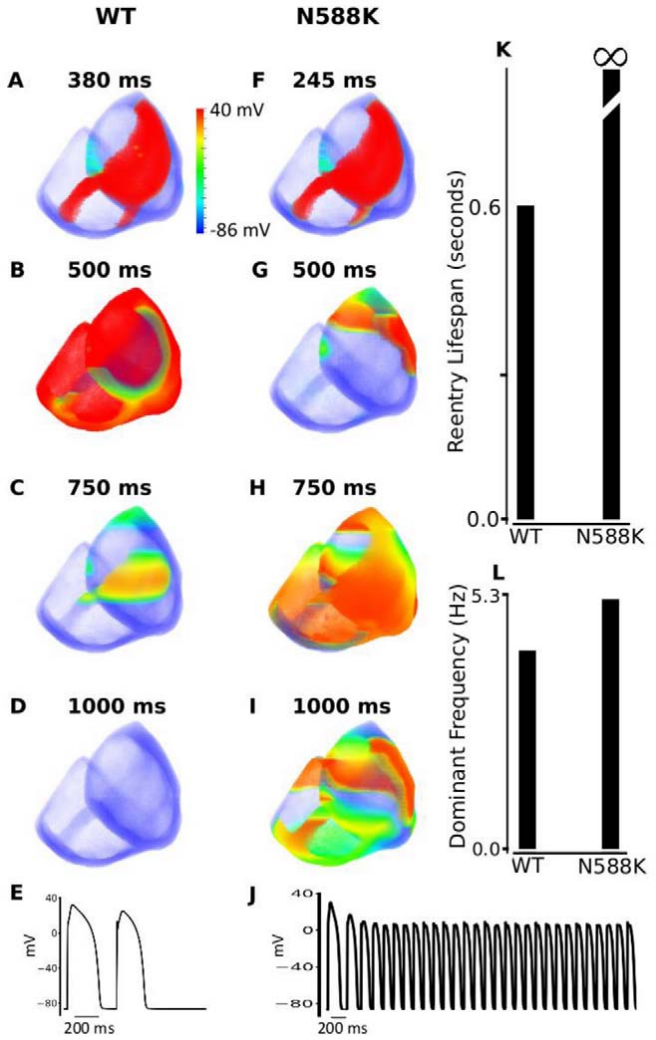
Snapshots of initiation and conduction of re-entry in a 3D anatomical model of human ventricles. (**A, F**) Application of a S2premature stimulus in a local region at refractory period of a previous conditioning excitation wave after a time delay of 380 ms for WT and 245 ms for N588K condition from the initial conditioning wave stimulus. (**B, G**) Developed scroll wave from the S2 stimulus. Snapshot at time = 500 ms. (**C, H**) Snapshot of scroll wave at time = 750 ms. The scroll wave self-terminated in WT, but persisted and broke up forming regenerative wavelets in N588K condition, (**D and I**) Snapshot of scroll wave at time = 1000 ms. Scroll wave self-terminated in WT before this recording point, but still persisted in N588K condition. (**E and J**) Evolution of the action potential of a cell in the left ventricle for WT and N588K conditions. (**K**) Measured lifespan of re-entry scroll wave in WT and in N588K condition. (**L**) Computed dominant frequency of electrical activity recorded from ventricle in WT and N588K conditions (2.7 Hz for WT and 5.3 Hz for N588K condition).

Results of 3D simulations are shown in Figure 12, which illustrate snapshots of evolution of re-entrant scroll waves (WT: Figure 12A–D; N588K mutation: Figure 12F–I) arising from a response to a premature stimulus applied to the basal region of the right ventricle, after a time delay of the initial excitation wave. Under the control condition, the excitation wave was first initiated in the apex region of the left ventricle. Once initiated, it propagates rapidly along the endocardial surface towards the base regions of the left and right ventricles, and then conducted transmurally across the ventricle wall (Figure 12A and Figure 12F). The activation conduction pattern and measured conduction time sequences in the 3D model of ventricles match to experimental data [29]. In the base of the right ventricle, a premature stimulus (with an amplitude of −104 pA/pF, size of 90 by 63 mm) was applied after a time delay of 380 ms for WT and 245 ms for N588K from the initial control wave stimulus. As the surrounding tissue at the stimulus site was in its relative refractory period, the premature stimulus evoked an excitation wave that propagated uni-directionally in the retrograde direction of the control excitation wave, leading to formation of a re-entrant scroll wave within the transmural wall (Figure 12B and Figure 12G). For the WT condition, the scroll wave self-terminated with a lifespan of 600 ms (Figure 12C & D). However, with the N588K mutation condition, the scroll wave broke up forming multiple wavelets that were sustained throughout the whole simulation period of 5 s (Figure 12H & I). Power spectrum analysis on the registered pseudo-ECG shows the dominant frequency of ventricle excitation to be approximately 2.7 Hz for the WT condition, and approximately 5.3 Hz for the N588K mutation condition (Figure 12L). Figures 12E and 12J show a recording of the evolution of the AP of a cell in the left ventricle for the WT and N588K conditions. Results with the S2 stimulus applied to a similarly sized sub-endocardial region are similar to the results (not shown) when the stimulus was applied epicardially. These 3D results concur with the 2D simulations, further illustrating the pro-arrhythmic effects of the N588K mutation.

## Discussion

### Summary of major findings

Due to the lack of a mammalian experimental model that can accurately reproduce the kinetic changes of SQTS mutations, ventricular cell and tissue models *in silico* provide a valuable means of investigating the functional effects of SQT mutations on genesis and maintenance of ventricular arrhythmias. The major findings of the present study are summarised as follows: (i) the Markov chain I_Kr_ formulations reproduce better the dynamic properties of hERG/I_Kr_ under both WT and N588K hERG SQT1 conditions than do the Luo-Rudy and TNNP H-H formulations; (ii) the N588K hERG mutation is causally linked to QT interval shortening, whether or not I_Kr_ is presumed to be homogeneously distributed across the ventricular wall; however, a heterogeneous I_Kr_ density across the ventricular strand TNNP model was found to be necessary to reproduce a taller T-wave amplitude as has been seen clinically in SQT1; (iii) with a heterogeneous I_Kr_ density across the strand, the N588K mutation leads to augmented membrane potential differences (δV) between paired cells and transmural APD dispersion in localised regions of the transmural strand that contributes to the increased T-wave amplitude; (iv) the N588K mutation increases at some localised regions the tissue's temporal vulnerability to the genesis of uni-directional conduction by a premature excitation; (v) the N588K mutation shortens the minimal tissue substrate size that facilitates the maintenance of re-entry as shown in both idealised and realistic tissue models of the human ventricle. These findings substantiate the causal link between the N588K mutation and QT interval shortening and, moreover, provide a comprehensive explanation for increased susceptibility to re-entry and perpetuation of re-entrant arrhythmia in the setting of SQT1.

### Significance of the study

Previous studies have used computer models to study the functional impact of the N588K hERG, KvLQT1 V307L (SQT 2) and KCNJ2 (SQT 3) mutations on ventricular cell AP shortening and characteristics of simulated ECGs [10], [18], [19]. However, whilst comprehensive investigation of the pro-arrhythmic effects of the V307L-KCNQ1 SQT 2 mutation using simplified multi-cellular geometry has been conducted before [19], simulations addressing the effects of the N588K mutation on perpetuating and facilitating re-entrant excitation waves in ventricular tissue have not been performed until now. Furthermore, the present study is the first to determine the arrhythmogenic consequences of the N588K mutation on multi-scale models of the human ventricles. In an earlier study by Kogan and colleagues [30], delayed outward K^+^ current deactivation rate was modified to determine effects on excitation-recovery and spiral wave activity. Slowing deactivation had a profound effect on wave-front propagation in those simulations. Whilst the results of that prior study are important in demonstrating a link between augmented K^+^ conductance (through deactivation modification) and arrhythmogenesis, the SQT1 I_Kr_ defect is principally one of impaired inactivation not deactivation and therefore requires targeted simulations that specifically reproduce I_Kr_ changes with the SQT1 N588K mutation. Prior studies [18], [20] have reported inhomogeneous shortening of ventricular APD with loss of I_Kr_ inactivation, which appears paradoxical in light of increased arrhythmia susceptibility in the syndrome. The present study resolves this apparent contradiction: whilst we have also demonstrated that the AP shortening as a result of N588K mutation is inhomogeneous, (resulting in a decreased APD_90_ and ERP dispersion, as the greatest shortening of APD_90_ occurred in MIDDLE cell APs), with heterogeneous I_Kr_ in the ventricle the mutation augmented both membrane potential difference between paired cells and the APD dispersion in some localised regions of the transmural strand. These changes lead to an increased T-wave amplitude, which is different to previous simulation results but is consistent with clinical observations [7], [25]–[27]. These changes also lead to increased tissue vulnerability to uni-directional conduction block in response to a premature stimulus. We have also shown that, in the setting of either a homogeneous or an inhomogeneous distribution of I_Kr_, the N588K mutation markedly reduced the minimal substrate size of the tissue essential for sustaining re-entrant excitation waves, enabling re-entrant excitation waves to persist in both idealised 2D and realistic 2D & 3D models. In all tissue models, an initial single re-entrant excitation wave may degenerate into multiple re-entrant wavelets, leading to a transition from tachycardia-like to fibrillation-like electrical excitation waves.

### Relevance to previous studies

Prior experimental work employing the canine perfused ventricular wedge preparation has used the K-ATP channel activator pinacidil to explore arrhythmogenesis in the setting of abbreviated repolarization [16]. Though mechanistically distinct from a hERG channel mutation, I_K,ATP_ activation by pinacidil was seen to lead to preferential mid-myocardial AP abbreviation, to increased dispersion of repolarization and to increased susceptibility to VT with programmed electrical stimulation [16]. Similar results were found subsequently using the I_Kr_ agonist PD-118057 and the wedge preparation [4]. Our data show for the first time that, in general terms, a similar substrate occurs *in silico* with biophysically accurate I_Kr_ models based on N588K-hERG. We have reported previously that the adult SQT 2 variant (incorporating the V307L-KCNQ1 mutation into the I_Ks_ channel) also involves heterogeneous APD abbreviation and increased refractory dispersion and susceptibility to genesis of re-entry in the ventricle [19]. However, the extent and nature of transmural APD dispersion produced by SQT 2 and SQT 1 appear to differ: whilst SQT 2 augments the APD dispersion across the whole transmural strand [19], SQT 1 only augments the APD dispersion in some localised regions (Figure 7F). We have found that with a heterogeneous I_Kr_ density across the ventricle wall, SQT1 may lead localised regions of tissue to have an augmented APD dispersion and membrane potential difference. These changes can account not only for an increased T-wave amplitude of the ECG as observed clinically, but can also produce increased tissue vulnerability to conduction block in localised regions of the ventricular wall, thereby facilitating re-entry. Our simulations suggest some similarity between SQT1 and SQT2 [19], however, in that both the N588K mutation (this study) and the V307L-KCNQ1 mutation [19] reduce the minimal size of ventricular tissue to sustain re-entrant activity.

### Limitations

Although the TNNP model has been derived from experimental data on the kinetics of ionic channels mostly obtained from human ventricular myocytes, has been validated by its ability to reproduce the experimental data from which the model was derived, and is suggested to be well-suited to the study of re-entrant arrhythmias in human ventricular tissue [31], [32], it has some limitations (discussed in detail elsewhere; [31], [32]). For example, due to the lack of complete experimental data sets on the transmural heterogeneity of human ion channel current densities, there are some discrepancies between the simulated transmural APD dispersion and those observed experimentally [33] - the model-generated ENDO APD tends to be close to that of the EPI APD. In addition, the model assumed a uniform I_Kr_ density across the ventricle wall, which may not be accurate as experimental data have shown a more abundant mRNA in the EPI region of the human ventricle [24]. Also, in the multicellular tissue model - due to a lack of detailed experimental data - the distance between contiguous regions of distinctive cell types and intercellular electrical coupling was chosen here to produce a positive T-wave and a conduction velocity of a planar solitary excitation wave close to the experimental data [34]. Nevertheless, it should be noted that the proportions of each sub-region used in our study are similar to those used in other studies [18], [20], [28], [35]. In the 2D “realistic” model, although we have considered intrinsic anisotropic intercellular electrical coupling, the complex anatomical structure of ventricular wall and fibre orientation, the 2D model is just based on a single slice of the ventricle wall, lacking consideration of the 3D anatomical structure and anisotropy, which could influence perpetuation of ventricular arrhythmias due to the N588K mutation. This limitation has been taken into account in our study design, however, through the incorporation of additional work using our ‘realistic’ 3D geometry model, the results of which also support a re-entrant substrate in SQT1. The fact that results from the combined use of 2D simplified and ‘realistic’ models and of a 3D ‘realistic’ geometry model in this study point towards similar underlying arrhythmogenic mechanisms suggests that, at least in the setting of SQT1, relatively simple 2D models appear adequate to provide mechanistic insight into arrhythmogenesis in this condition. It remains to be determined, through a similar multi-level simulation approach, whether this is the case for other forms of SQT. It should be noted that other possible contributory mechanisms for the pro-arrhythmia in SQT1 have also been proposed. These include the generation of early EADs [17] and an augmented dispersion of action potential repolarization between the Purkinje fibre network and the ventricle in the SQT1 context [11], [13]. Additional single cell simulations (not shown) did not support the occurrence of early EADs in our model, however, whilst further studies are warranted in the future to incorporate biophysically and anatomically accurate Purkinje fibre cell networks into 2D and 3D models. Nevertheless, whilst it is important that potential limitations of the models used are made explicit, these limitations do not influence fundamentally the conclusions that can be drawn on likely mechanisms by which the N588K hERG mutation facilitates arrhythmia induction and maintenance.

### Conclusions

On the basis of the simulations in this study it can be concluded that not only is the N588K mutation causally linked to QT interval shortening, but also that it leads to increased APD dispersion and difference in membrane potential (δV) in some localized regions, that increase tissue vulnerability to the genesis of re-entry by a premature excitation. Moreover, the mutation shortens tissue ERP that facilitates the maintenance of re-entry in both 2D and 3D tissue scenarios. Thus, the findings of this study provide a comprehensive explanation for clinical consequences of this form of the SQTS in terms of abbreviation of repolarization and susceptibility to arrhythmia. The multiscale ventricular models developed and employed in this study may have further utility for probing the basis of arrhythmia in other forms of the SQTS and other repolarization disorders.

## Methods

### Development of I_Kr_ Markov chain model

The full and reduced Markov chain model formulations for I_hERG_/I_Kr_ formulation were based on the work of Kiehn *et al.* [36], Clancy and Rudy [37], Lu *et al.* [21] and Wang *et al.* [38]. The models were modified to incorporate the experimentally-observed kinetic properties of WT and N588K-mutated hERG/I_Kr_ channel. These kinetic properties include (1) the profound (> +60 mV) shift in the voltage dependence of inactivation of N588K-hERG that alters rectification of I_hERG_ [7], [12], [13], [39]; (2) the substantial increase of I_hERG_ early during the ventricular action potential (AP) waveform [7], [12], [13], [39]; and (3) the generation of rapid, transient, outward currents in response to premature, depolarizing stimuli under ‘paired stimuli’ experiments [13].

A schematic illustration for the full Markov chain model is shown in Figure 1Ai. It consists of three closed states (C1, C2 and C3), an open state (O) and an inactivated state (I). Inactivation can occur from either the open or closed state but does so preferentially from the open state [40]. From the full Markov chain model, a reduced Markov chain model (r-MC) was created by removing the transitions from the closed state (C3) to the inactivated state (I) and vice-versa (Figure 1Aii). Consequently, inactivation occurs only from the open state in such a model [38].

To obtain the transition rates of the Markov chain model that reproduced the experimentally-observed kinetic properties of WT and N588K-hERG/I_Kr_, we simulated the experimental current-voltage (I-V) relationships for WT and N588K-hERG/I_Kr_ using the voltage clamp protocol from McPate *et al.* [12], [13]. The membrane potential was held at −80 mV and then depolarized briefly to −40 mV (to evaluate instantaneous current), followed by 2 s depolarisations to a range of potentials from −40 mV to +60 mV (in 10 mV increments); finally, tail currents were elicited by repolarization to −40 mV for 4 s.

Variables that modified these transition rates were introduced and their values were obtained by minimising the least-squared difference between the experimental data and the simulation. The variables that produced the best fit and behaviour of macroscopic currents relative to the experimental data were selected (see Text S1). The minimisation was performed using the Broyden-Fletcher-Goldfarb-Shanno (BFGS) algorithm [41].

### AP clamp

The Markov chain model formulations were validated by comparing simulated results from different voltage clamp protocols – premature stimuli (Figure 2Aiii–Eiii) and AP Clamp (Figure 2) – to experimentally obtained data. To simulate the AP clamp, the same digitised ventricular AP used to generate the experimental data [13] was used to elicit the I_hERG_ in the simulation. For paired premature stimuli simulations, the protocol utilising paired ventricular AP waveform commands was applied [13]. Following an initial ventricular AP command, a second, premature AP command waveform was superimposed 100 ms before the APD_90_ (action potential duration at 90% repolarization) of the first command. The premature stimulus was then applied in 10 ms increments for subsequent sweeps [13].

### Single cell model and AP simulations

The hERG/I_Kr_ Markov chain model formulations were then incorporated into the ten Tusscher, Noble, Noble and Panfilov (TNNP) human ventricular cell model [32]. The model reproduces human ventricular cell and membrane channel properties and it reproduces transmural heterogeneity of the AP [31], [32]. It has also been suggested to be suited to the study of re-entrant arrhythmias in human ventricular tissue [31], [32]. In 2006, based on newly available experimental data, Xia *et al* [42] updated and modified the TNNP model, which was employed in the present study.

In the single cell model, the cell membrane is modelled as a capacitor connected in parallel with variable resistances and batteries representing the different ionic channel, exchange and pump currents. Hence, the electrophysiological behaviour of a cell can be described with the following differential equation:
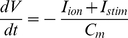
(1)where *V* is voltage, *t* is time, *I_ion_* is the sum of all transmembrane ionic currents, *I_stim_* is the externally applied stimulus current and *C_m_* is the cell capacitance per unit surface area.

*Eq. 1* was integrated using the forward Euler method with a time step of 0.02 ms. The Hodgkin-Huxley-type equations for the gating variables of the various time-dependent currents in the TNNP model were integrated using the Rush and Larsen scheme [43] while the I_Kr_ Markov chain model was integrated with the forward Euler method.

### Measurement of APD

Action potential duration (APD) was defined as the action potential duration at 90% repolarization (APD_90_). APs were elicited with an S1–S2 protocol consisting of 10 S1 stimuli and an S2 stimulus. The S1 stimuli were applied at a frequency of 1 Hz and twice the strength of the threshold value. The S2 was applied at some diastolic interval (DI) after the AP evoked by the last S1 stimulus. The Action Potential Duration Restitution (APD-R) curve was generated by decreasing the DIs and plotting the APD_90_ evoked by the S2 stimulus against the DIs. Steady-state rate dependence of the APD curve was determined by pacing single cell models at different basic cycle lengths (BCL) and plotting the APD_90_ against the BCLs. At varying BCLs, the Effective Refractory Period (ERP) was measured as the smallest DI for which the overshoot of the AP evoked by the S2 stimulus reached 80% of the AP evoked by the 10th S1 stimulus at each PCL. The Effective Refractory Period restitution (ERP-R) curve was generated by plotting the measured ERP against BCLs [44].

### Other I_Kr_ models used for comparison

We compared results from the I_Kr_ Markov chain model to three other I_Kr_ models, including (i) the reduced Markov model (r-Mc); (ii) the original I_Kr_ formulation of the TNNP model; and (iii) the I_Kr_ formulation from the Luo Rudy model [45], [46].

This comparative approach is similar to and complements recent work from Bett *et al.* [47] who have recently compared WT Markov and HH formulations for hERG, describing qualitative and quantitative differences that influence the predictive properties of the different models studied [47]. The TNNP and Luo-Rudy I_Kr_ formulations models are Hodgkin-Huxley type models.

The TNNP I_Kr_ formulation [31], [32] is described by:
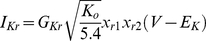
(2)where *x_r1_* is an activation gate and *x_r2_* is an inactivation gate. *G_kr_* is the maximal conductance of I_Kr_ and is set to 0.153 nS pF^−1^ for both WT and N588K-hERG, *K_O_* is the extracellular K^+^ concentration, 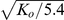 represents the *K_O_* dependence of the current, *V* is the membrane potential and *E_K_* is the K^+^ reversal potential given by the Nernst equation. To enable the TNNP formulation reproduce our N588K-hERG experimental data, the steady state value of the activation gating variable was doubled. The original formulation, without modification, served as the WT formulation.

The Luo-Rudy I_Kr_ formulation is described by:
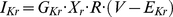
(3)where *X_r_* is a time-dependent activation gate and *R* is a time-dependent inactivation gate. *G_kr_* is the maximal conductance of I_Kr_ and is modelled as 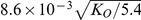 nS pF^−1^ for both WT and N588K-hERG. *V* is the membrane potential and *E_Kr_* is the K^+^ reversal potential given by the Nernst equation. To enable the Luo-Rudy formulation reproduce our N588K-hERG experimental data, ‘*R*’ was set to a value of 1 for mimicking defective inactivation with the mutation. The original formulation, without modification served as the WT formulation.

### Heterogeneous transmural ventricular tissue model

Initiation and conduction of action potentials in multicellular tissue models was modelled with the monodomain equation [48], [49]:
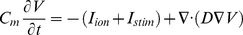
(4)where *D* is the diffusion coefficient describing the tissue conductivity.

For one-dimensional (1D) computations, the mesh used was a single fibre, 15 mm long with 100 nodes that were spaced 0.15 mm apart, with each node representing a 150-µm cylindrical cell. This total strand length of 15 mm is consistent with the normal range of human transmural ventricle width; ∼4.0–14.0 mm [50]–[52]. In the strand, there were 25 endocardial cells (ENDO), 35 middle cells (MCELL) and 40 epicardial cells (EPI). The corresponding lengths of each region were 3.75 mm for ENDO, 5.25 mm for MCELL and 6 mm for EPI. The chosen proportion for each region is similar to those used in other studies [18]–[20], [28].

The diffusion coefficient, ‘*D*’ was set at 0.001 cm^2^/ms giving a planar conduction velocity of 65 cm/s through the strand. This is close to the 70 cm/s velocity of conduction along the fibre direction in human myocardium [34]. ‘*D*’ was homogenous except for a 5-fold decrease at the EPI-MCELL border [19], [28].

To initiate a conducting excitation wave, a supra-threshold stimulus was applied at the ENDO end of the strand. The conduction velocity was calculated from nodes one-quarter and three-quarters of the way along the strand. The activation time of each point was defined to be the time at which the maximum upstroke velocity occurred.

In two-dimensions (2D) simulations, both idealised and realistic geometries were implemented. The idealised geometry was a simple sheet of tissue measuring 15 mm by 50 mm. It was modelled by expanding the 1D transmural strand (length of 15 mm in the *x*-direction) into a sheet with a width of 50 m in the *y*-direction.

The realistic geometry was a transverse cross-sectional slice taken from the middle of a 3D ventricular geometry reconstructed by DT-MRI [35] with a spatial resolution of 0.2 mm. It was segmented into distinctive regions of endo-, middle and epi-cardiac layers with similar contiguous figurations in the transmural wall as in the 1D strand model. It also implemented anisotropic fibre orientations as used in the work of [35]. The intracellular conductivities in the fibre and cross-fibre directions were set to 0.3 and 0.1 mS mm^−1^ respectively.

In three-dimensions (3D), simulations were performed using an anatomical human ventricle geometry of a healthy 30 year-old male that was reconstructed by using DT-MRI. It has a spatial resolution of 0.2 mm with approximately 24.2 million nodes in total and includes anisotropic fibre orientation. The tissue was segmented into distinctive ENDO, MIDDLE and EPI regions for both the left and right ventricles [29]. The conditional activation sites were determined empirically across the ventricles walls, and were validated by reproducing the activation sequence and the QRS complex in the measured 64-channel ECG [29] of that person. In simulations, the intercellular conductivities in the fibre, cross-fibre and sheet directions were set to 0.3, 0.1 and 0.31525 mS mm^−1^ respectively.

### Numerical methods

For the 1D, 2D and 3D simulations, equation 4 was solved using a Strang splitting scheme [53] and a Crank-Nicholson time-stepping scheme in the temporal direction, together with Lagrangian Q1 finite elements in the spatial direction using the deal.II adaptive finite element library [54]. The Strang splitting scheme is second-order accurate and the Crank-Nicholson time-stepping scheme is unconditionally stable and second-order accurate with respect to time [55]. The resulting computed solution is therefore second-order accurate.

The system of linear algebraic equations resulting from the discretisation of the monodomain equation was solved using the preconditioned Conjugate Gradient method with the Symmetric Successive OverRelaxation (SSOR) method as the preconditioner [56].

The TNNP single cell model was converted to CUDA/C++ via the Thrust CUDA library [57]. The cell kinetics are represented by a system of ODEs (I_ion_ in equation 4). In the 1D, 2D and 3D simulations, the collection of systems of ODEs (I_ion_) for all the cells was solved on a Tesla C2050 “Fermi” GPU with 448 CUDA cores. The host system for the Tesla GPU is a Dell Precision T7500 with 12 Intel Xeon CPU cores at 2.80 GHz and 96 GB of memory.

### Computing the pseudo-ECG

The pseudo-ECG was computed following the method of Gima and Rudy [28]. At the extracellular space located at position (*x′, y′, z′*), a far-field unipolar potential can be computed as an integral of the spatial gradient of membrane potential at position (*x,y,z*) on the strand by:
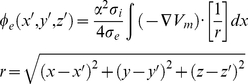
(5)where *σ_e_* and *σ_i_* are the extracellular and intracellular conductivities respectively, *α* is the radius of the strand, *r* the distance from a source point (*x,y,z*) to a field point (*x′,y′,z′*). The pseudo-ECG was computed as 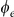 at a position 2.0 cm away from the epicardial end of the strand.

### Initiation of re-entry in 2D sheet

In both the regular and realistic geometries, re-entry was initiated by a standard S1–S2 protocol. In the regular, idealised 2D sheet, a plane wave was initiated at the ENDO end by an S1 stimulus. During the vulnerable window of the tissue, an S2 stimulus was applied to a local tissue area in the EPI region to evoke unidirectional propagation that can lead to re-entry.

### Measurement of minimal size of S2 that sustains re-entry in 2D models

Corresponding to a S2 stimulus, unidirectional conduction of the S2-evoked excitation wave leads to formation of a paired re-entrant excitation waves, with their tips counter-rotates with each other. If the distance between the two tips is sufficient long, each of the two tips has sufficient space to complete its pathway, and therefore, the paired re-entrant excitation waves sustain. Otherwise, the two tips collide leading to termination of the re-entrant excitation wave. To provide an adequate re-entrant pathway, a sufficient S2 size is required, which is dependent on the wavelength of the spiral wave. In order to evaluate the critical size of re-entrant pathway of tissue, we estimated the minimal spatial S2 length that supported the formation of re-entrant spiral waves under control and SQT1 conditions. This minimal length of S2 gives an indication of the susceptibility of the tissue to re-entry, i.e., the larger the minimal length, the harder the initiation of re-entry.

### Initiation of re-entry in 2D heart cross-section

In the 2D realistic model of a cross-sectional slice, multiple stimulus sites were chosen in an effort to recreate the activation pattern observed by Durrer *et al.* [58]. To initiate re-entry, an S2 stimulus was applied in the endocardium of the left ventricle (see Figure 11) partly during the repolarization phase of a conditioning wave and partly within fully recovered tissue. The S2-evoked excitation wave propagates uni-directionally, leading to formation of re-entrant excitation wave within the transmural wall (Figure 11).

### Initiation of re-entry in the 3D anatomical human ventricles

3D scroll waves were initiated by using an S1–S2 protocol. The S1 stimulus was applied to multi-stimulation sites that produced the activation timing sequence across the ventricles as seen experimentally. The S2 stimulus was applied over a small epicardial region consisting mainly of the left ventricle and a fraction of the right-ventricular outflow tract (see Figure 12) during the refractory tail of the S1 stimulus.

## Supporting Information

Video S1 **WT reentry in idealised 2D geometry.** Initiation and conduction of re-entry in a 2D idealised model of the transmural ventricle under the wild type (control) condition. A planar conditioning wave generated by an S1 stimulus at the ENDO end propagates towards the EPI end. An S2 stimulus is applied to the EPI part during the vulnerable window of the local tissue at 356 ms, which develops into a spiral wave. The spiral wave self-terminates within 1000 ms.
(MP4)

Video S2 **N588K reentry in idealised 2D geometry.** Initiation and conduction of re-entry in a 2D idealised model of the transmural ventricle under the N588K (SQT1 mutant) condition. A planar conditioning wave generated by an S1 stimulus at the ENDO end propagates towards the EPI end. An S2 stimulus is applied to the EPI part during the vulnerable window of the local tissue at 226 ms, which develops into a spiral wave. The spiral wave persists under the SQT1 condition.
(MP4)

Video S3 **WT reentry in realistic 2D geometry.** Re-entrant spiral waves generated by the application of a premature S2 stimulus into the refractory and partially recovered region of an excitation wave after a delay of 400 ms rom the initial wave stimulus under the wild type (control) condition. The induced spiral waves transition from transmural re-entry with tip rotating within the ventricle wall to anatomical re-entry with tip rotating around the ventricle boundary and self-terminate within 2000 ms.
(MP4)

Video S4 **N588K reentry in realistic 2D geometry.** Re-entrant spiral waves generated by the application of a premature S2 stimulus into the refractory and partially recovered region of an excitation wave after a delay of 241 ms rom the initial wave stimulus under the N588K (SQT1 mutant) condition. The induced spiral waves transition from transmural re-entry with tip rotating within the ventricle wall to anatomical re-entry with tip rotating around the ventricle boundary. The spiral waves persist under the SQT1 condition and break up forming regenerative multiple re-entrant wavelets.
(MP4)

Video S5 **WT reentry in realistic 3D geometry (anterior view).** Anterior view of the ventricles showing re-entrant scroll waves generated by the application of a premature S2 stimulus in a local refractory region of a previous conditioning excitation wave after a time delay of 380 ms from the initial conditioning wave stimulus under the wild type (control) condition. The scroll waves self-terminate within 1000 ms.
(MP4)

Video S6 **WT reentry in realistic 3D geometry (left ventricular view).** Left ventricular view of the ventricles showing re-entrant scroll waves generated by the application of a premature S2 stimulus in a local refractory region of a previous conditioning excitation wave after a time delay of 380 ms from the initial conditioning wave stimulus under the wild type (control) condition. The scroll waves self-terminate within 1000 ms.
(MP4)

Video S7 **N588K reentry in realistic 3D geometry (anterior view).** Anterior view of the ventricles showing re-entrant scroll waves generated by the application of a premature S2 stimulus in a local refractory region of a previous conditioning excitation wave after a time delay of 245 ms from the initial conditioning wave stimulus under the N588K (SQT1 mutation) condition. The scroll waves self-terminate within 1000 ms.
(MP4)

Video S8 **N588K reentry in realistic 3D geometry (left ventricular view).** Left ventricular view of the ventricles showing re-entrant scroll waves generated by the application of a premature S2 stimulus in a local refractory region of a previous conditioning excitation wave after a time delay of 245 ms from the initial conditioning wave stimulus under the N588K (SQT1 mutation) condition. The scroll waves persisted and broke up forming regenerative wavelets.
(MP4)

Text S1 **Appendix.** Appendix showing the: (i) I_Kr_ Markov model equations for WT and N588K conditions (ii) concordance between simulations and experimental currents elicited by action potential waveforms (iii) steady state APD rate dependence for the full Markov Chain model and (iv) model independence of the functional consequences of the N588K mutation for cell models.
(DOC)
