# Supplementary material for: Increased Vulnerability of Human Ventricle to Re-entrant Excitation in hERG-linked Variant 1 Short QT Syndrome
Source: PLoS Comput Biol. 2011 Dec 15;7(12):e1002313. doi: 10.1371/journal.pcbi.1002313 (PMC3240585; doi:10.1371/journal.pcbi.1002313)
Supplement: Text S1 — Appendix. Appendix showing the: (i) IKr Markov model equations for WT and N588K conditions (ii) concordance between simulations and experimental currents elicited by action potential waveforms (iii) steady state APD rate dependence for the full Markov Chain model and (iv) model independence of the functional consequences of the N588K mutation for cell models. (DOC) [file pcbi.1002313.s009.doc]

**Supporting Text S1**

***I_Kr_* MODEL EQUATIONS**

$$I_{Kr}=G_{Kr}\times O_{Kr}\times\left(V_{m}-E_{Kr}\right)$$

*O_Kr_* is the open probability

$G_{Kr}=0.0243\times\left[K^{+}\right]_{o}^{0.59}$ (Unit: nS/pF)

$$E_{Kr}=\frac{RT}{F}\log\frac{\left[K^{+}\right]_{o}}{\left[K^{+}\right]_{i}}$$

**RATE TRANSITION EQUATIONS**

**WT**

$\alpha_{1}=2.172$ (1)

$\beta_{1}=1.077$ (2)

$\alpha_{2}=0.00655\times e^{0.027735765\times\left(V-36\right)}$ (3)

$\beta_{2}=0.001908205\times e^{-0.0148902V}$ (4)

$\alpha_{i}=0.04829\times e^{-0.039984\times\left(V+25\right)}\times\left(\frac{4.5}{K_{O}}\right)$ (5)

$\beta_{i}=0.2624\times e^{\left(0.000942V\right)}\times\left(\frac{4.5}{K_{O}}\right)^{0.3}$ (6)

$\alpha=0.00555\times e^{0.05547153\left(V-12\right)}$ (7)

$\beta=0.002357\times e^{-0.036588V}$ (8)

$\mu=\frac{\alpha_{i}\beta_{2}}{\beta_{i}}$ (9)

**RATE TRANSITION EQUATIONS**

**N588K**

$\alpha_{1}=2.172$ (1)

$\beta_{1}=0.5385$ (2)

$\alpha_{2}=0.001965\times e^{0.05547153\times\left(V-21\right)}$ (3)

$\beta_{2}=2.260489\times10^{-6}\times e^{-0.0925782V}$ (4)

$\alpha_{i}=0.439\times e^{-0.02352\times\left(V+40\right)}\times\left(\frac{4.5}{K_{O}}\right)$ (5)

$\beta_{i}=0.0164\times e^{0.000942\times\left(V+15\right)}\times\left(\frac{4.5}{K_{O}}\right)^{0.3}$ (6)

$\alpha=0.00555\times e^{0.05547153\left(V+3\right)}$ (7)

$\beta=0.002357\times e^{-0.036588V}$ (8)

$\mu=\frac{\alpha_{i}\beta_{2}}{\beta_{i}}$ (9)

**Simulating I_Kr_ currents – concordance between simulations and experimental currents elicited by action potential waveforms**

During conventional voltage clamp simulations (Figure 2Ai and Bi of main paper text), whilst experimental and simulated (f-MC and r-MC) I-V relations were similar, during the elicited currents an initial transient WT I_hERG_ current was observed on step depolarization that was not evident in published experimental recordings [1-2]. It is notable that Bett *et al.* [3] have recently made a study of five Markov models (including that on which our f-MC model is based [4]) and their ability to reproduce experimental data. It was found that in the initial activation phase, due to rapid inactivation following activation, a transient current is produced as the initial response to depolarisation. All the Markov models showed, to varying degrees, this feature. In optimising our model formulations, our principal focus was that the models should be able to reproduce WT and SQT1 hERG current profiles during action potential (AP) voltage clamp (i.e. during physiological waveforms). The advantage of the AP clamp technique over a conventional voltage clamp is its ability to record ionic currents during dynamic, physiological waveforms, therefore facilitating the recording of currents that are influenced by membrane potential history [5-6]. Our WT and N588K model Markov formulations were found to reproduce well the current profile during an action potential (AP) clamp (Figure S1) and were able also to reproduce experimental data obtained with paired AP clamp protocols (Figure 2, main text). Differences between WT current in AP and step voltage-clamp in respect of the presence/absence of a transient current component on initial depolarization are likely to reflect the differing extents and nature (step change versus dynamically changing) of membrane potential depolarisation during conventional versus AP clamp protocols. For comparison, Figure S2 shows the results of trying to reproduce the same experimental data under AP clamp using ten Tusscher-Noble-Noble-Panfilov (TNNP) Hodgkin-Huxley (H-H) I_Kr_ formulation [7].

**
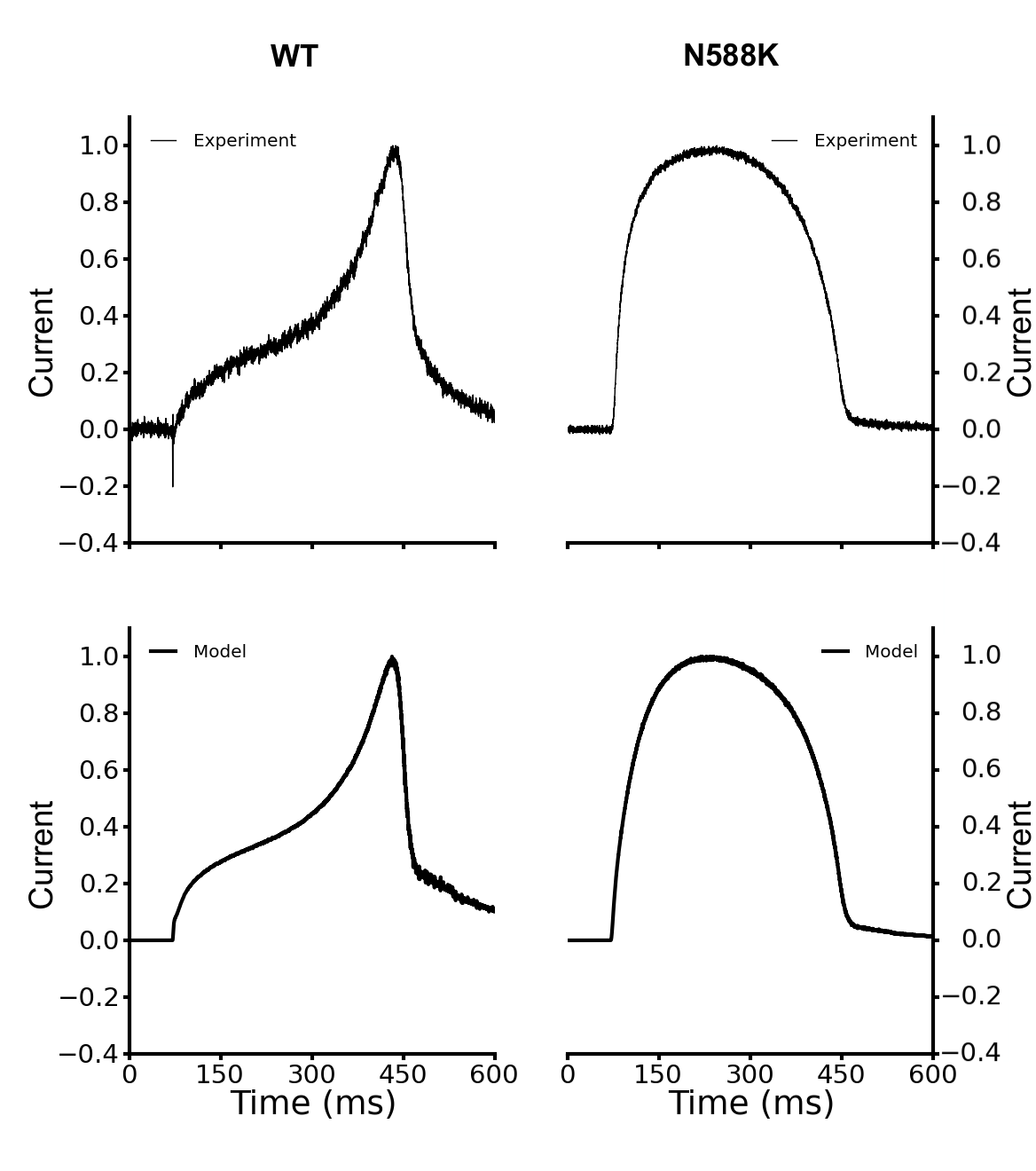
**

**Figure S1.** Profile of I_hERG_ during ventricular AP voltage command under WT and N588K

conditions obtained with the full Markov Chain model (f-MC).


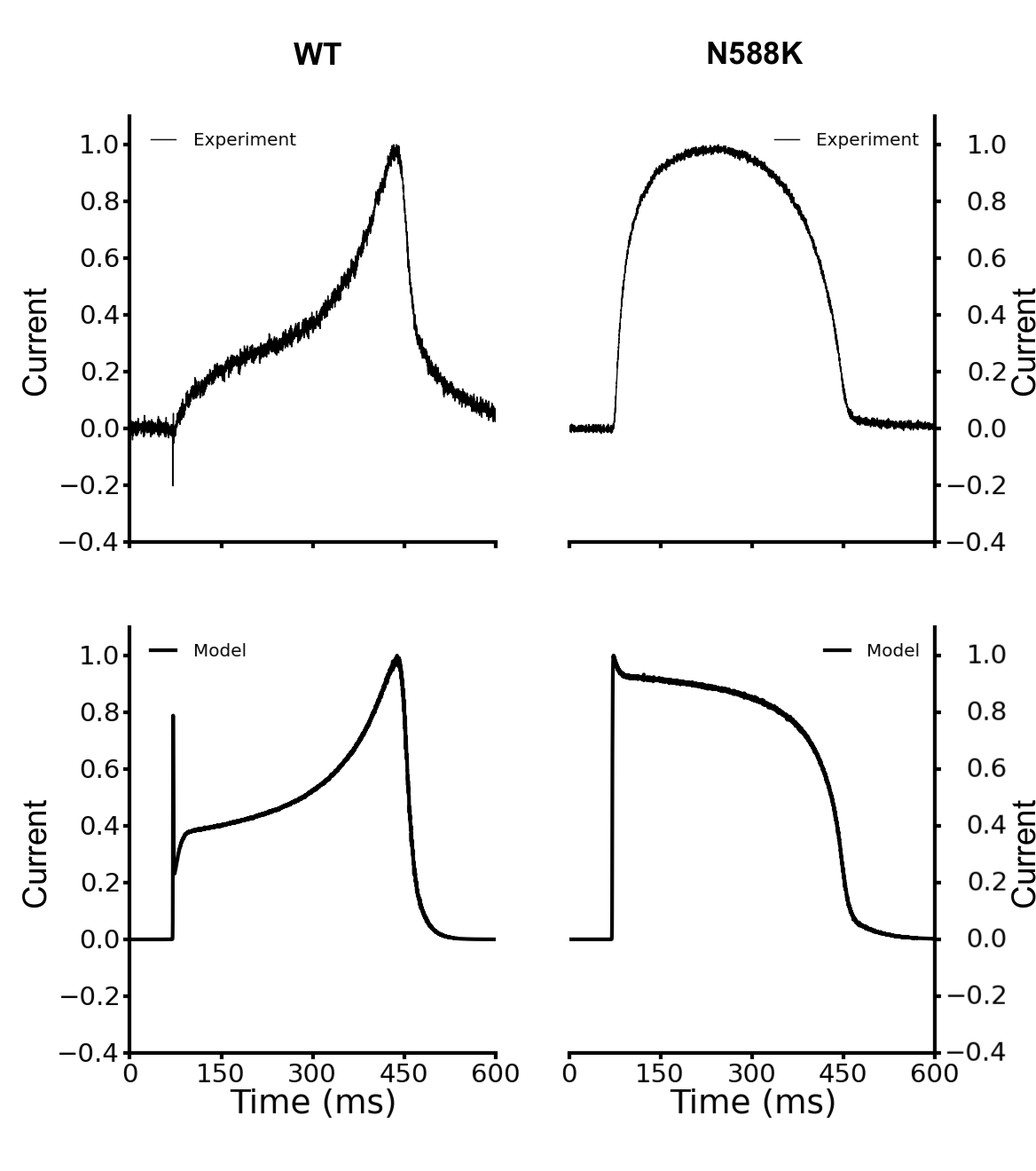


**Figure S2.** Profile of I_hERG_ during ventricular AP voltage command under WT and N588K conditions obtained with the original TNNP I_Kr_ formulation model.

**Steady State APD Rate Dependence for the Full Markov Chain Model**

With the f-MC model incorporated into the TNNP ventricular cell model [7], we also investigated the effect of the N588K mutation on steady state rate dependence in the different cell types compared to the WT condition. The result is shown in Figure S3.

**
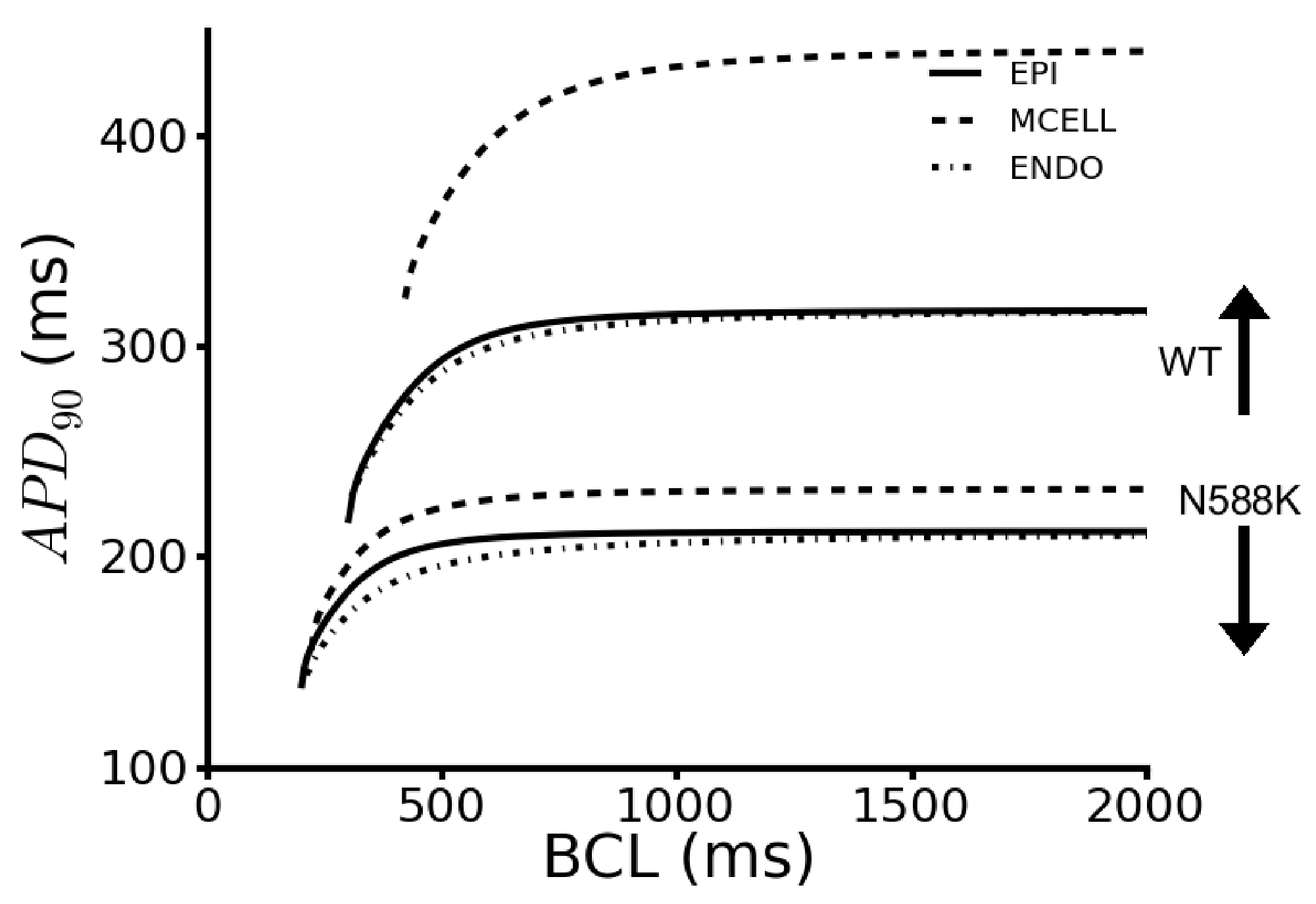
**

**Figure S3.** Steady state APD rate dependence for the Full Markov Chain model (f-MC) incorporated into the TNNP ventricular action potential cell model.

**Model independence of the functional consequences of the N588K mutation for cell models**

In addition to using the TNNP ventricular cell model [7], we have used the Grandi-Pasqualini-Bers (GPB) [8] and O'Hara-Rudy dynamic (ORd) [9] human ventricular cell models to test the single cell predictions of our N588K mutation model. The data obtained with these models support our original results; APD heterogeneity is decreased, APD restitution is flattened (Figure S4) and ERP is decreased (Figure S5). Consequently, these effects of the SQT1 N588K-hERG mutation are model independent.


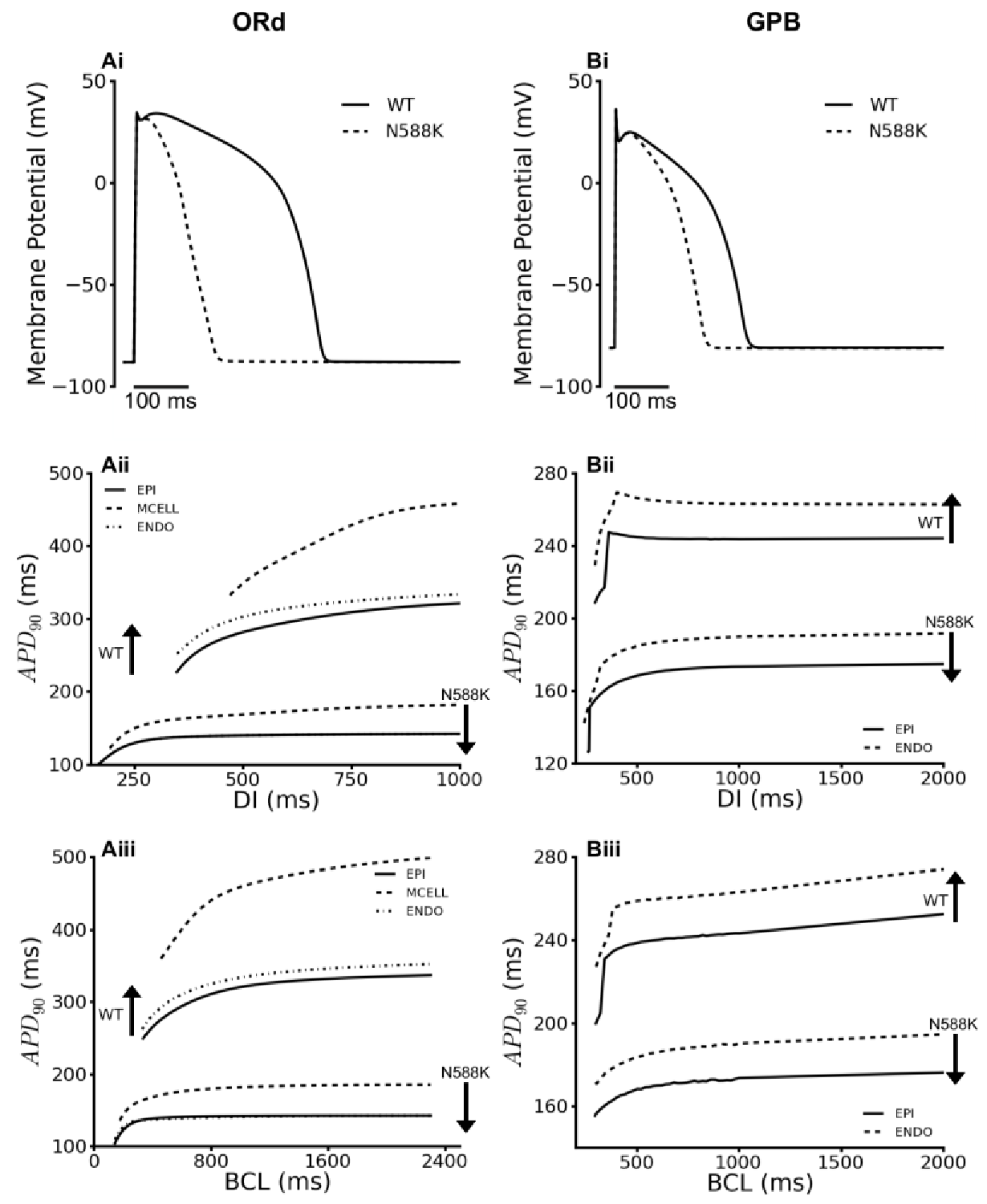


**Figure S4. Simulation of action potential and rate-dependent APD restitution curves for the O’Hara-Rudy dynamic (ORd) and the Grandi-Pasqualini-Bers (GPB) human ventricular cell models**

**(Ai, Bi)** Steady state (1 Hz) epicardial action potentials using the ORd and GPB human ventricular cell models.

**(Aii, Bii)** WT and N588K APD restitution curves for EPI, MIDDLE and ENDO cells respectively for the ORd and GPB models (the latter lacks MIDDLE CELLS).

**(Aiii, Biii)** WT and N588K steady state APD rate dependence for EPI, MIDDLE and ENDO cells respectively for the ORd and GPB models (the latter lacks MIDDLE CELLS).

**
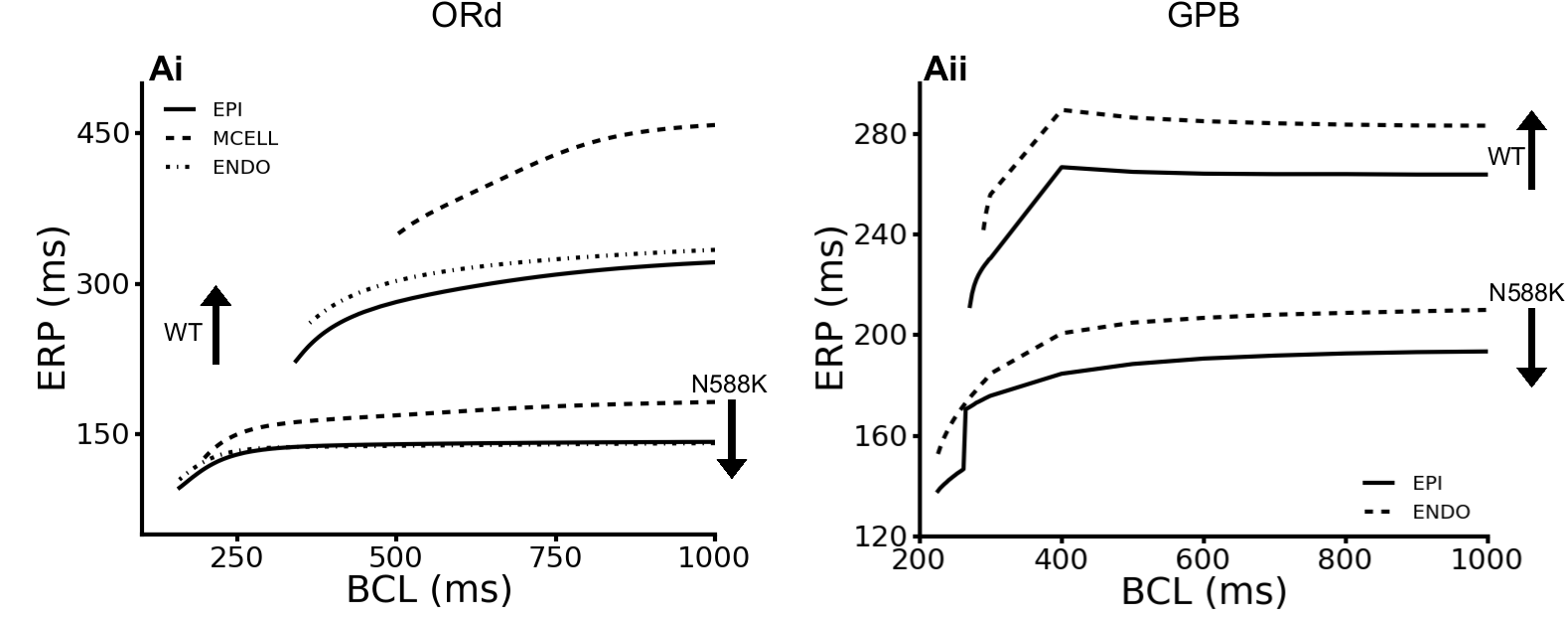
**

**Figure S5. Simulation of effective refractory period (ERP) restitution curves for the O’Hara-Rudy dynamic (ORd) and the Grandi-Pasqualini-Bers (GPB) human ventricular cell models**

**(Ai)** WT and N588K ERP restitution curves for EPI, MIDDLE and ENDO cells respectively for the ORd.

**(Aii)** WT and N588K ERP restitution curves for EPI, MIDDLE and ENDO cells respectively for the GPB models (it lacks MIDDLE CELLS).

**Heterogeneous I_Kr_ distribution is necessary to produce an upright T-wave in SQT1**

Using the TNNP model, I_Kr_ heterogeneity (and associated increase in δv) was necessary to produce an increased T-wave amplitude in the setting of the N588K mutation. In additional simulations using the ORd model (Figure S6: Ai-Aiv), at an EPI:MID ratio of 1:1, although the T wave of the pseudo-ECG was positive under the WT condition (Figure S5: Ai), it became inverted in the SQT1-mutation condition, This is due to the N588K mutation completely removing the APD heterogeneity between EPI and ENDO cells of the ORd model, resulting in an inverted T-wave when I_Kr_ was similar in the two cell types. However, at increasing EPI:MID I_Kr_ ratios, which re-confer APD heterogeneity on these cell types, the T-wave amplitudes increased and become positive (Figure S6: Aii-Aiv). The critical EPI:ENDO ratio at which the T-wave transitioned from the inverted to positive position is 1.4:1. It is important to note that the effect of mutant I_Kr_ channel on APD heterogeneity was so large that even at the higher EPI:MID ratios, the T-wave amplitude was not tall and peaked as seen on SQT1 patient ECGs or in our simulations with the TNNP model.

Simulations with the GPB model also confirmed the prerequisite for transmural I_Kr_ heterogeneity to enable a positive T-wave in the SQT1 setting (Figure S6: Bi – Biv). The GPB model only considers the EPI and ENDO cell types and ignores the MID cell type. Using this model we observed that in the WT condition, although the EPI and ENDO heterogeneity in the APD is small, it was sufficient to produce a positive T-wave. However, in the N588K mutation setting, in the absence of I_Kr_ heterogeneity, transmural APD heterogeneity was abolished, producing an inverted T-wave. The degree of inversion was reduced as EPI:ENDO I_Kr_ ratio was increased; when the I_Kr_ EPI-ENDO ratio was set to > 1.5:1, part of the T-wave started to become slightly positive.


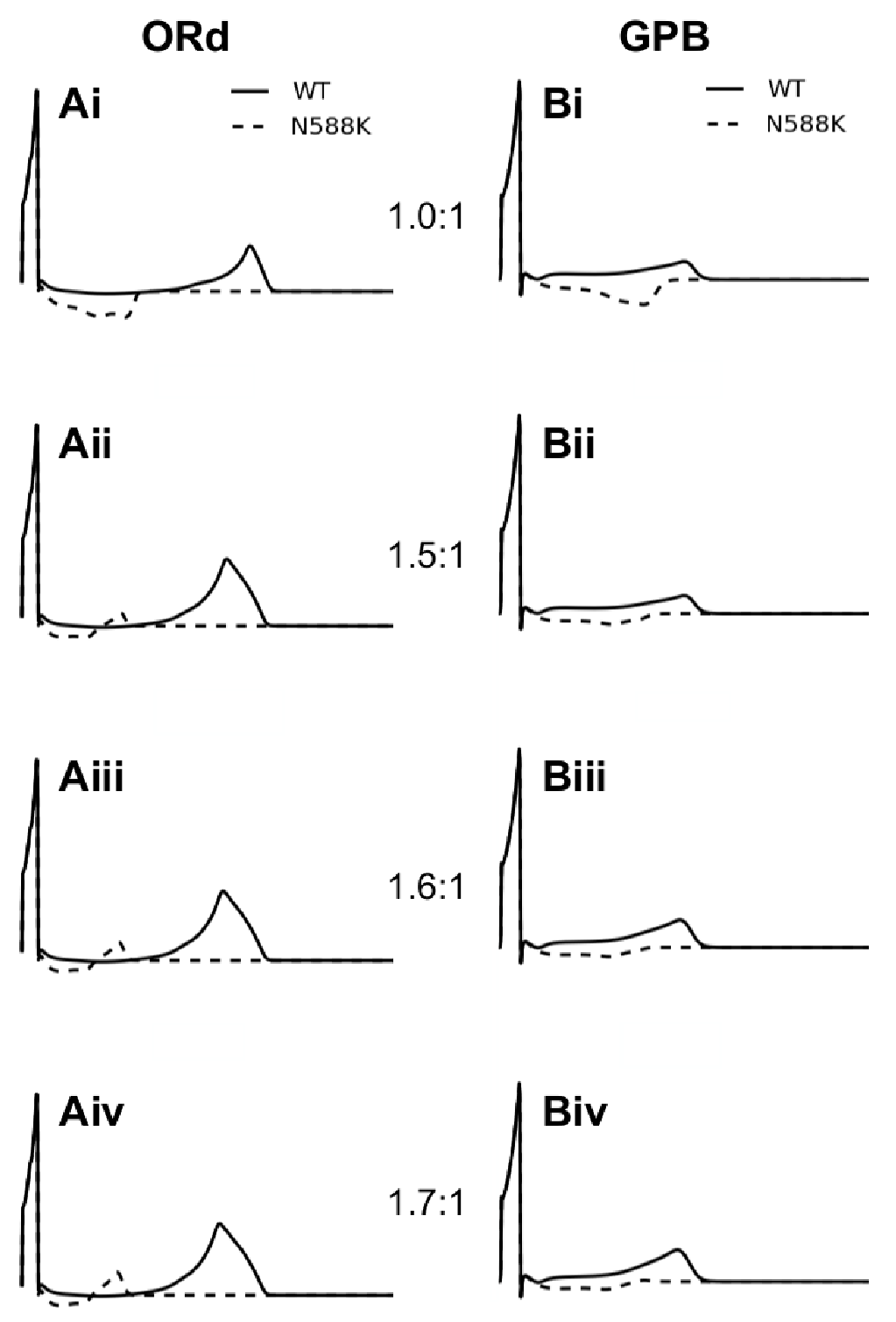


**Figure S6. Computed pseudo-ECGs using the O’Hara-Rudy dynamic (ORd) and the Grandi-Pasqualini-Bers (GPB) human ventricular cell models**

**(Ai, Bi)** Pseudo-ECGs corresponding to the WT (thick line) and N588K (dashed line) conditions respectively with a 1.0:1 EPI to MID (ORd) or EPI-ENDO (GPB) ratio.

**(Aii, Bii)** Pseudo-ECGs corresponding to the WT (thick line) and N588K (dashed line) conditions respectively with a 1.5:1 EPI to MID (ORd) or EPI-ENDO (GPB) ratio.

**(Aiii, Biii)** Pseudo-ECGs corresponding to the WT (thick line) and N588K (dashed line) conditions respectively with a 1.6:1 EPI to MID (ORd) or EPI-ENDO (GPB) ratio.

**(Aiv, Biv)** Pseudo-ECGs corresponding to the WT (thick line) and N588K (dashed line) conditions respectively with a 1.7:1 EPI to MID (ORd) or EPI-ENDO (GPB) ratio.

**References**

1. McPate MJ, Duncan RS, Milnes JT, Witchel HJ, Hancox JC (2005) The N588K-HERG K^+^ channel mutation in the 'short QT syndrome': mechanism of gain-in-function determined at 37°C. Biochem Biophys Res Commun 334: 441-449.
2. McPate MJ, Duncan RS, Hancox JC, Witchel HJ (2008) Pharmacology of the short QT syndrome N588K-hERG K^+^ channel mutation: differential impact on selected class I and class III antiarrhythmic drugs. Br J Pharmacol 155: 957-966.ten Tusscher KHWJ, Panfilov AV (2006) Alternans and spiral breakup in a human ventricular tissue model. Am J Physiol Heart Circ Physiol 291: H1088-H1100.
3. Bett GCL, Zhou Q, Rasmusson RL (2011) Models of HERG Gating. Biophys J 101: 631-642.
4. Clancy CE and Rudy Y (2001) Cellular consequences of HERG mutations in the long QT syndrome: precursors to sudden cardiac death. Cardiovasc Res 50: 301-313.
5. Noble D, Varghese A, Kohl P, Noble P (1998) Improved guinea-pig ventricular cell model incorporating a diadic space, I_Kr_ and I_Ks_, and length- and tension-dependent processes. Can J Cardiol 14: 123-134.
6. Hancox JC, Levi AJ, Witchel HJ (1998) Time course and voltage dependence of expressed HERG current compared with native "rapid" delayed rectifier K current during the cardiac ventricular action potential. Pflugers Arch 436: 843-853.
7. ten Tusscher KHWJ, Panfilov AV (2006) Alternans and spiral breakup in a human ventricular tissue model. Am J Physiol Heart Circ Physiol 291: H1088-H1100.
8. Grandi E, Pasqualini FS, Bers DM (2010) A novel computational model of the human ventricular action potential and Ca transient. J Mol Cell Cardiol 48: 112-121.
9. O'Hara T, Virag L, Varro A, Rudy Y (2011) Simulation of the undiseased human cardiac ventricular action potential: model formulation and experimental validation. PLoS Comput Biol 7: e1002061.
